# Asymmetric total synthesis of smyrindiol employing an organocatalytic aldol key step

**DOI:** 10.3762/bjoc.8.123

**Published:** 2012-07-18

**Authors:** Dieter Enders, Jeanne Fronert, Tom Bisschops, Florian Boeck

**Affiliations:** 1 Institute of Organic Chemistry, RWTH Aachen University, Landoltweg 1, 52074 Aachen, Germany

**Keywords:** aldol reaction, asymmetric synthesis, organocatalysis, proline, smyrindiol

## Abstract

The first organocatalytic asymmetric synthesis of smyrindiol, by using an (*S*)-proline catalyzed enantioselective intramolecular aldol reaction as the key step, is described. Smyrindiol was synthesized from commercially available 2,4-dihydroxybenzaldehyde in 15 steps, with excellent stereoselectivity (de = 99%, ee = 99%). In the course of this total synthesis a new and mild coumarin assembly was developed.

## Introduction

**Figure 1:**
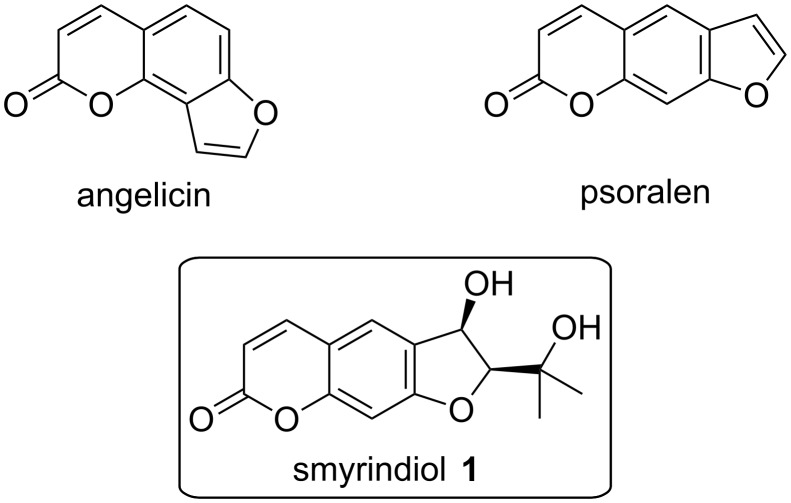
Furocoumarins.

Furocoumarins are a group of compounds that are structurally derived from psoralen or angelicin (Figure 1) [1]. Naturally occurring furocoumarins are mainly found in plants of the Apiaceae and Rutaceae families [2] and are used in the treatment of vitiligo, psoriasis and other skin diseases [3]. Some furocoumarins exhibit vasodilatory, antifungal and antibacterial activity [4–5].

Smyrindiol (**1**), also called (+)-(2’*S*,3’*R*)-3-hydroxymarmesin [7], is a linear dihydrofurocoumarin, which was isolated from the roots of Smyrniopsis aucheri by Dzhafarov et al. in 1992 [6] and from the roots of Brosimum gaudichaudii by Vilegas et al. in 1993 [7]. Its 1'-*O*-glucoside was isolated in 1982 by Lemmich et al. from the roots of Angelica archangelica [4]. Smyrindiol has shown antifungal and antibacterial effects [5].

**Scheme 1:**
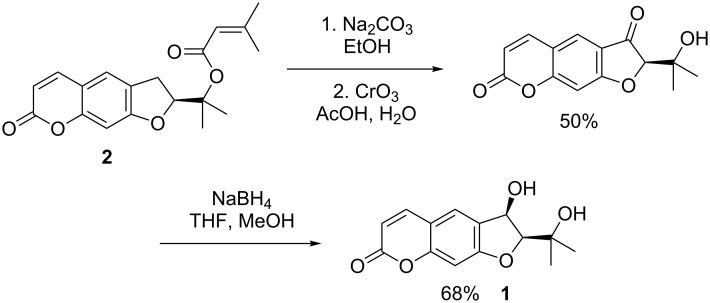
Synthesis of smyrindiol (**1**) by Grande et al.

The first synthesis of smyrindiol was described by the group of Grande [8]. Starting with the naturally occurring dihydrofurocoumarin (−)-prantschimgin (**2**), the hydroxy group in 3-position was introduced by a Cr(VI)-mediated benzylic oxidation, followed by a diastereoselective sodium borohydride reduction (Scheme 1).

**Scheme 2:**
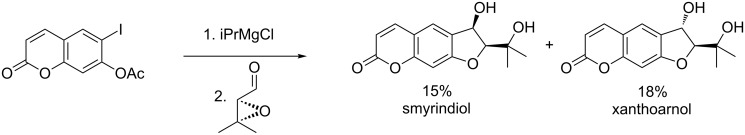
Synthesis of smyrindiol by Snider et al.

A few years later Snider et al. described the, up to now, sole total synthesis of smyrindiol using an enantiomerically pure epoxy aldehyde [9]. The two possible diastereoisomers resulting from the addition of the epoxy aldehyde to the coumaryl Grignard intermediate, occurred with low diastereoselectivity, with a slight preference (18% versus 15%) towards the *anti*-isomer xanthoarnol (Scheme 2).We now wish to present the first diastereo- and enantioselective, organocatalytic, asymmetric synthesis of smyrindiol.

## Results and Discussion

### Retrosynthetic analysis

**Scheme 3:**
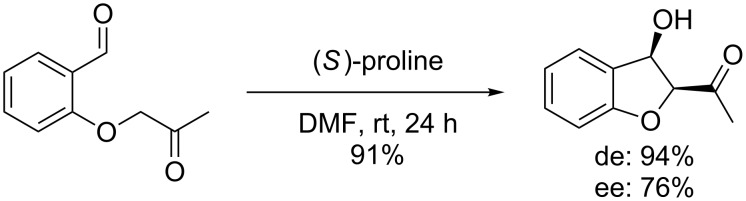
Proline-catalyzed intramolecular aldol reaction of *O*-acetonyl-salicylaldehydes.

Previously the proline-catalyzed intramolecular aldol reaction of *O*-acetonyl-salicylaldehydes was described by our research group (Scheme 3) [10].

**Scheme 4:**
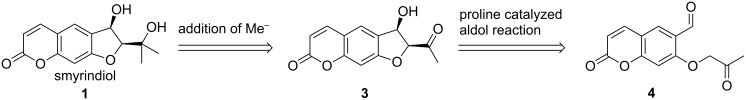
First retrosynthetic analysis.

We envisaged that this 5-*enolexo* aldolization could be utilized to construct the 1,3-diol moiety of smyrindiol, if a coumarin derivative of the *O*-acetonyl-salicylaldehyde were to be used. The addition of a methyl group to the carbonyl of the aldol product would yield smyrindiol (Scheme 4).

**Scheme 5:**
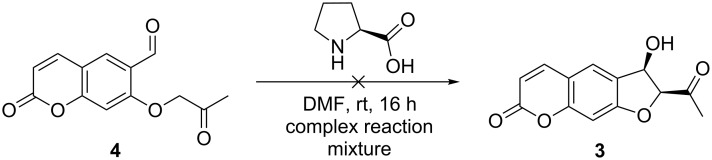
Attempted proline catalyzed aldol reaction.

Unfortunately, all attempts to conduct the intramolecular aldol reaction with the ketoaldehyde **4** failed (Scheme 5). Instead of the expected hydroxy ketone **3**, we obtained a complex reaction mixture. This was probably the result of unwanted intermolecular side reactions of the keto group of one molecule on the coumarin system of another molecule, as an NMR analysis of the reaction mixture showed that an aldehyde signal was still present after the reaction and had, thus, not taken part in the aldol reaction. For this reason, we decided to construct the coumarin system *after* the proline catalyzed aldol reaction had taken place.

**Scheme 6:**
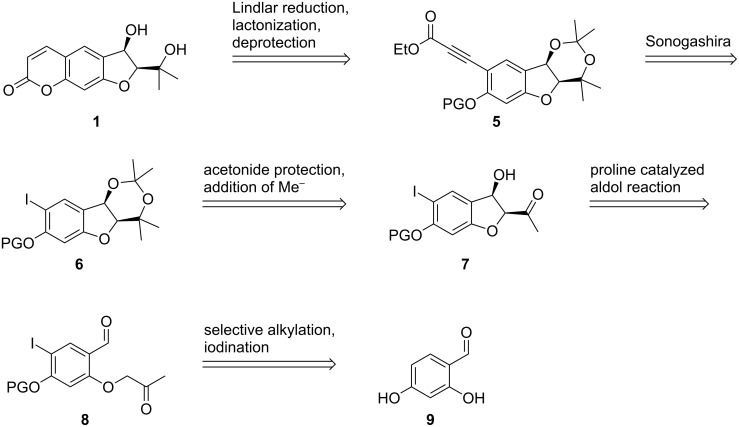
Second retrosynthetic analysis.

We envisaged that the preparation of the coumarin system of smyrindiol (**1**) could be achieved by Lindlar reduction of the propiolate ester **5**, in which the 1,3-diol would be protected as an acetonide, whereupon lactonization would take place simultaneously. Alkyne **5** could be synthesized through a Sonogashira coupling with iodide **6**. This acetonide could be formed by addition of a methyl anion equivalent to the carbonyl group of the aldol product **7** with subsequent protection of the 1,3-diol. The substrate for the aldol key step **8** could be prepared starting with commercially available 2,4-dihydroxybenzaldehyde (**9**) by a selective iodination of the 5-position of the aromatic ring, protection of the hydroxy group in the 4-position, and 2-*O*-acetonylation of the selectively protected salicylaldehyde (Scheme 6).

**Scheme 7:**
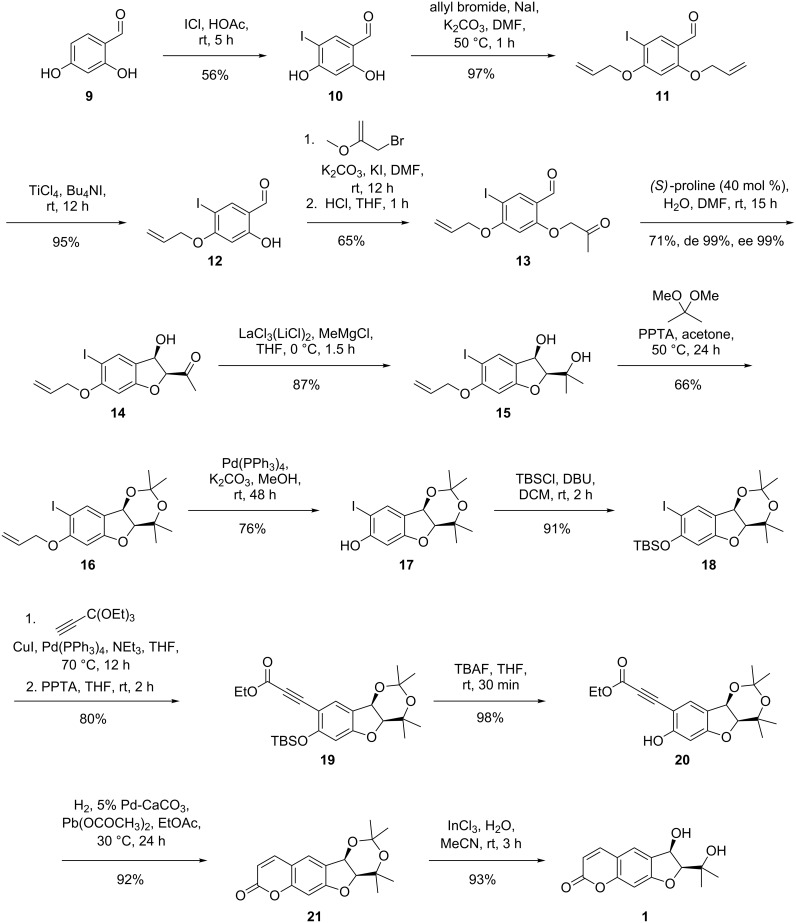
Asymmetric total synthesis of smyrindiol (**1**).

The total synthesis of smyrindiol is depicted in Scheme 7. Firstly, the *O*-acetonyl-salicylaldehyde **13**, as the substrate for the aldol reaction, was synthesized in five steps. Iodination of commercially available 2,4-dihydroxybenzaldehyde (**9**) with iodine monochloride in acetic acid [11] furnished a mixture of different iodination products. After the addition of water to the reaction mixture, only the 5-iodo derivative **10** precipitated from the solution and could be separated from the other isomers by filtration in 56% yield. We decided to protect the 4-hydroxy group as an allyl ether, as we found that other protecting groups were either not compatible with the reaction conditions towards acetonide **15** or were found to be impossible to cleave afterwards while leaving the rest of the molecule intact.

Since we were unable to conduct a selective mono-allylation of the hydroxy group in the 4-position, both hydroxy groups were allylated, followed by a selective, titanium tetrachloride/tetra-*n*-butylammonium iodide mediated deprotection of the salicylic hydroxy group. The selectivity of this deprotection is caused by the chelating effect of the carbonyl oxygen towards titanium [12], directing the Lewis acidic species to the oxygen in the 2-position. In spite of this circuitous protection–deprotection procedure, the yield is excellent (92% over two steps) and the procedure is straightforward. The mono-allylated phenol must then be converted to the *O*-acetonylaldehyde **13**.

The alkylation of the monoprotected salicylaldehyde **12** with 1-bromo- or chloroacetone under basic conditions led to side products due to a subsequent base-catalyzed aldol reaction. For this reason, the alkylation was conducted under basic conditions with 2-methoxyallyl bromide as a masked acetonylating reagent. Hydrolysis of the methyl vinyl ether with dilute acid liberated the ketone derivative **13**. The synthesis of this starting material for the envisaged intramolecular aldol reaction could be conducted on a multigram scale without the need for column chromatography.

Similar to the previously reported aldol reaction (Scheme 3), the (*S*)*-*proline catalyzed 5-*enolexo* aldol key step of this synthesis could be performed in good yield (71%). To our delight, we found in this case an exceedingly high diastereo- (99%) and enantioselectivity (99%) for the aldol reaction, furnishing the aldol product **14** as a single stereoisomer.

The introduction of the methyl group by using the Imamoto protocol [13] employing cerium(III) chloride and methyllithium at −78 °C, was unreliable with highly varying yields of the 1,3-diol. This irreproducibility is probably due to the heterogeneous nature of the reaction mixture. Fortunately, the use of Knochel's published modification [14] of this reaction using a lanthanum(III) chloride bis(lithium chloride) complex solution and a methyl Grignard reagent proved to be robust and produced the desired 1,3-diol **15** in high yields (87%). The 1,3-diol was found to be sensitive towards condensation to the benzofuran system; thus, for further modifications it was protected as acetonide **16** in a moderate yield (66%) using 2,2-dimethoxypropane under PPTA catalysis.

As we turned towards the Sonogashira reaction of the iodide **16** with a propiolic acid derivative, we found that the allyl protecting group was cleaved readily by the palladium present in the reaction mixture. Since the Sonogashira reaction did not occur with the unprotected *ortho*-iodophenol **17**, we decided to reprotect the phenol as a *tert*-butyldimethylsilyl (TBS) ether, using TBS chloride and DBU. Reprotection was necessary, since employing the TBS protected ketoaldehyde **8** (PG = TBS) in the proline catalyzed aldol reaction led to complete condensation to the corresponding benzofuran.

Propiolic acid esters are known to be problematic substrates for Sonogashira reactions, due to side reactions [15]. For this reason, we used an orthoester, which coupled smoothly with iodide **18** and could be transformed into the aryl alkynoate **19** under mild acidic conditions, leaving the acetonide unscathed. The TBS group could now be cleaved by the addition of tetrabutylammonium fluoride in THF. When subjected to Lindlar catalyst and 1 atm of hydrogen, the resulting ethyl (2-hydroxyphenyl)propiolate **20** could be reduced to the corresponding *ortho*-hydroxy-(*Z*)-cinnamate, which ring closed immediately to coumarin **21**. Deprotection of the acetonide under acidic conditions proved to be difficult due to the acid labile nature of the 1,3-diol. However, indium(III) catalysis in acetonitril in the presence of water was found to cleave the acetonide selectively, yielding smyrindiol (**1**), whose spectroscopic data were identical to those published in the literature [9].

## Conclusion

In summary, we have developed the first asymmetric organocatalytic total synthesis of smyrindiol, using an (*S*)*-*proline catalyzed 5-*enolexo* aldol reaction as the key step. The diastereo- and enantioselectivity is virtually complete (de 99%, ee 99%), and the title compound was obtained in 15 steps in an overall yield of 6.3%. All steps were performed under mild conditions with short reaction times. Our novel total synthesis should allow the synthesis of larger quantities of the natural compound without having to rely on natural sources. Needless to mention, the unnatural enantiomer could be synthesized if (*R*)-proline were to be used as the organocatalyst. In addition, the Sonogashira/Lindlar reduction/lactonization sequence opens a new efficient and flexible entry to the coumarin core of other natural products.

## Supporting Information

File 1 Experimental procedures and characterization of compounds.

File 2 NMR-spectra and chromatograms.
