# Supplementary material for: Asymmetric total synthesis of smyrindiol employing an organocatalytic aldol key step
Source: Beilstein J Org Chem. 2012 Jul 18;8:1112–7. doi: 10.3762/bjoc.8.123 (PMC3458728; doi:10.3762/bjoc.8.123)
Supplement: File 1 — Experimental procedures and characterization of compounds. [file Beilstein_J_Org_Chem-08-1112-s001.pdf]

Supporting Information File 1

for

Asymmetric total synthesis of smyrindiol employing an organocatalytic aldol key step

Dieter Enders*, Jeanne Fronert, Tom Bisschops and Florian Boeck

Address: Institute of Organic Chemistry, RWTH Aachen University, Landoltweg 1, 52074 Aachen, Germany

Email: Dieter Enders - enders@rwth-aachen.de

*Corresponding author

Experimental procedures and characterization of compounds

General: All reactions with air- and water-sensitive substances were carried out under argon in flame-dried glassware. Unless otherwise noted, all commercially available compounds were used without further purification. Tetrahydrofuran was freshly distilled under argon from Solvona® (Na on molecular sieve). Methanol was freshly distilled under argon over calcium chloride. For preparative column chromatography SIL G-25 UV_{254} from Macherey-Nagel, particle size 0.040–0.063 mm (230–240 mesh, flash) was used. Visualization of the developed TLC plates was performed with ultraviolet irradiation (254 nm). Optical rotation values were measured on a Perkin-Elmer 241 polarimeter. Microanalyses were performed with a Vario EL element analyzer. Mass spectra were measured on a Finnigan SSQ7000 (EI 70 eV)

spectrometer and high-resolution mass spectra on a Thermo Fisher Scientific Orbitrap XL. IR spectra were recorded on a Perkin-Elmer FT–IR Spectrum 100 using an ATR-Unit. ^{1}H and ^{13}C NMR spectra were recorded at ambient temperature on Varian Mercury 300, Inova 400, Varian VNMRS-400 or Varian VNMRS-600 spectrometers with tetramethylsilane as an internal standard. Analytical HPLC was performed on a Hewlett-Packard 1100 Series instrument using a chiral stationary phase (Daicel AD).

2,4-Dihydroxy-5-iodobenzaldehyde (10)

2,4-Dihydroxybenzaldehyde (**9**) (15.0 g, 109 mmol, 1.0 equiv) was dissolved in acetic acid (75 mL) under warming. At room temperature a solution of iodine monochloride (18.5 g, 114 mmol, 1.05 equiv) in acetic acid (20 mL) was added dropwise. After being stirred for an additional 5 h, the mixture was poured into water (250 mL) and a saturated sodium thiosulfate solution (50 mL). The solid was separated by filtration, washed with water and dried under reduced pressure. The product was obtained as a colorless solid (16.0 g, 56%).

R_f = 0.59 (pentane/EtOAc 2/1); mp 167–171 °C; ^{1}H NMR (400 MHz, DMSO) δ 11.56 (s, 1H), 10.95 (s, 1H), 9.91 (s, 1H), 7.95 (s, 1H), 6.53 (s, 1H) ppm; ^{13}C NMR (101 MHz, DMSO) δ 189.6, 163.7, 163.2, 140.7, 118.2, 102.6, 74.6 ppm; IR (ATR): $\tilde{\nu}$ = 3130, 3054, 2859, 2703, 2545, 2327, 2119, 2055, 1989, 1947, 1757, 1601, 1500, 1451, 1406, 1312, 1191, 1129, 1009, 885, 859, 831, 711, 680 cm^{-1}; MS (EI, 70 eV): *m/z* (%) = 263.9 (M^+, 100), 69.1 (15), 53.2 (27); anal. calcd. for $C_7H_5IO_3$: C 31.84, H 1.91; found: C 32.11, H 1.74.

2,4-Bis(allyloxy)-5-iodobenzaldehyde (11)

2,4-Dihydroxy-5-iodobenzaldehyde (**10**) (10.0 g, 37.9 mmol, 1.0 equiv), potassium carbonate (11.0 g, 80.0 mmol, 2.1 equiv), sodium iodide (0.6 g, 3.8 mmol, 0.1 equiv) and allylbromide (11.5 g, 95.0 mmol, 2.5 equiv) were dissolved in DMF (100 mL, 0.38 M) and stirred for 1 h at 50 °C. The solution was poured into a mixture of diethylether (140 mL) and water (140 mL). After extraction with diethylether (2 × 70 mL), the combined organic phases were washed with a solution of lithium chloride (5%, 4 × 30 mL) and a saturated sodium chloride solution (70 mL). After drying over magnesium sulfate, the solvent was removed under reduced pressure. The product was obtained as a colorless solid (12.7 g, 97%).

R_f = 0.69 (pentane/EtOAc 2/1); mp 115–116 °C; ^{1}H NMR (300 MHz, $CDCl_3$) δ 10.24 (s, 1H), 8.23 (s, 1H), 6.38 (s, 1H), 5.97–6.13 (m, 2H), 5.33–5.59 (m, 4H), 4.60–4.67 (m, 4H) ppm; ^{13}C NMR (75 MHz, $CDCl_3$) δ 186.9, 162.9, 162.6, 139.2, 131.9, 131.5, 120.7, 118.5, 118.4, 97.3, 76.4, 70.0, 69.5 ppm; IR (ATR): $\tilde{\nu}$ = 3084, 3019, 2989, 2851, 2761, 2330, 2116, 1893, 1797, 1659, 1583, 1462, 1407, 1369, 1314, 1262, 1199, 1148, 1099, 1035, 993, 924, 901, 855, 817, 761, 684 cm^{-1}; MS (EI, 70 eV): *m/z* (%) = 344.0 (M^+, 100), 217.2 (14), 203.2 (11), 175.1 (20), 148.2 (14), 147.2 (17), 91.2 (14); anal. calcd. for $C_{13}H_{13}IO_3$: C 45.37, H 3.81; found: C 45.42, H 3.42.

4-(Allyloxy)-2-hydroxy-5-iodobenzaldehyde (12)

At 0 °C titanium tetrachloride (13.9 g, 73.2 mmol, 2.0 equiv) was poured into DCM (210 mL, 0.17 M) followed by tetrabutylammonium iodide (27.0 g, 73.2 mmol, 2.0 equiv). After stirring for 15 minutes 2,4-bis(allyloxy)-5-iodobenzaldehyde (**11**) (12.6 g, 36.6 mmol, 1.0 equiv) was added. After another 15 minutes the reaction mixture was warmed to room temperature and stirred for 12 h. The solution was poured into a mixture of ice (30 g), a saturated solution of sodium hydrogen sulfate

(30 mL), hydrochloric acid (1 M, 30 mL) and diethylether (60 mL). The aqueous phase was extracted with diethylether (3 x 50 mL), and the combined organic phases were washed successively with hydrochloric acid (1 M, 50 mL), sodium bicarbonate solution (5%, 50 mL), sodium thiosulfate solution (0.1 M, 50 mL) and sodium-EDTA solution (5%, 20 mL). After being dried over magnesium sulfate, the solvent was removed under reduced pressure, and the residue was filtered over silica gel (pentane/diethylether 3/1) to give a colorless solid (10.6 g, 95%).

R_f = 0.44 (pentane/diethylether 3/1); mp 60–62 °C; ^{1}H NMR (400 MHz, $CDCl_3$) δ 11.41 (s, 1H), 9.67 (s, 1H), 7.88 (s, 1H), 6.38 (s, 1H), 5.99–6.10 (m, 1H), 5.54 (dd, 1H, *J* = 17.3 Hz, 1.5 Hz), 5.36 (dd, 1H, *J* = 10.6 Hz, 1.5 Hz), 4.64 (d, 2H, *J* = 5.0 Hz) ppm; ^{13}C NMR (101 MHz, $CDCl_3$) δ 193.5, 164.4, 163.4, 143.8, 131.2, 118.6, 117.1, 100.6, 74.3, 70.1 ppm; IR (ATR): $\tilde{\nu}$ = 2956, 2840, 2727, 2323, 2111, 1992, 1893, 1810, 1765, 1683, 1617, 1563, 1479, 1408, 1364, 1330, 1264, 1198, 1023, 981, 938, 887, 839, 769, 693 cm^{-1}; MS (EI, 70 eV): *m/z* (%) = 303.9 (M^+, 100), 177.1 (61), 163.1 (13), 121.2 (13), 52.5 (11), 51.5 (10); anal. calcd. for $C_{10}H_9IO_3$: C 39.50, H 2.98; found: C 39.61, H 2.88.

4-(Allyloxy)-5-iodo-2-(2-oxopropoxy)benzaldehyde (13)

4-(Allyloxy)-2-hydroxy-5-iodobenzaldehyde (**12**) (10.0 g, 32.9 mmol, 1.0 equiv), potassium carbonate (9.1 g, 65.8 mmol, 2.0 equiv), and potassium iodide (0.6 g, 3.3 mmol, 0.1 equiv) were stirred in DMF (50 mL, 0.7 M). A solution of 3-bromo-2-methoxyprop-1-ene (74.5 g, 10 wt%, 1.5 equiv) was added and the reaction mixture was stirred for 12 h at room temperature. Diethylether (40 mL) and a saturated ammonium chloride solution (30 mL) were added. The aqueous phase was extracted with diethylether (3 x 30 mL) and the combined organic phases were dried over sodium sulfate. After the solvent was removed under reduced pressure, the residue

was dissolved in THF and furnished with hydrochloric acid (0.5 mL, 1 M). After being stirred at room temperature for 1 h, the reaction was quenched with sodium bicarbonate solution (10 mL, 5%), the aqueous phase was extracted with diethylether (2 × 30 mL) and the combined organic phases were dried over sodium sulfate. After the solvent was removed under reduced pressure, the yellowish solid was dissolved in diethylether (20 mL) and furnished with pentane (40 mL). The precipitate was separated by filtration and dried under reduced pressure. The product was obtained as a light-yellow solid (7.70 g, 65%).

R_f = 0.15 (pentane/EtOAc 2/1); mp 140–142 °C; ^{1}H NMR (400 MHz, $CDCl_3$) δ 10.29 (s, 1H), 8.26 (s, 1H), 6.22 (s, 1H), 5.96–6.10 (m, 1H), 5.52 (dd, 1H, *J* = 17.3 Hz, 1.4 Hz), 5.38 (dd, 1H, *J* = 10.6 Hz, 1.4 Hz), 4.64 (d, 2H, *J* = 5.0 Hz), 4.63 (s, 2H), 2.33 (s, 3H) ppm; ^{13}C NMR (101 MHz, $CDCl_3$) δ 203.8, 186.4, 162.7, 161.7, 139.8, 131.4, 120.5, 118.6, 96.9, 77.5, 73.3, 70.1, 26.8 ppm; IR (ATR): $\tilde{\nu}$ = 3101, 2908, 2865, 2771, 2325, 2127, 2000, 1942, 1853, 1805, 1731, 1661, 1579, 1483, 1407, 1354, 1314, 1262, 1210, 1172, 1152, 1102, 1067, 1027, 984, 923, 904, 836, 682 cm^{-1}; MS (EI, 70 eV): *m/z* (%) = 360.0 (M^+, 100), 303.0 (79), 302.0 (45), 233.2 (64), 177.1 (22), 175.2 (47), 150.2 (21), 149.2 (23), 148.2 (28), 147.2 (41), 133.2 (24), 131.2 (21), 121.2 (79), 119.2 (28), 103.2 (18), 91.3 (34), 79.3 (23), 69.3 (26), 63.3 (75), 57.3 (57), 51.4 (36); anal. calcd. for $C_{13}H_{13}IO_4$: C 43.35, H 3.64; found: C 43.79, H 3.67.

1-((2*S*,3*R*)-6-(Allyloxy)-3-hydroxy-5-iodo-2,3-dihydrobenzofuran-2-yl)ethanone (14)

4-(Allyloxy)-5-iodo-2-(2-oxopropoxy)benzaldehyde (**13**) (1.20 g, 3.32 mmol, 1.0 equiv) was dissolved in DMF (13.3 mL, 0.25 M). *(S)*-Proline (0.15 g, 1.33 mmol, 0.40 equiv) and water (0.06 g, 3.32 mmol, 1.0 equiv) were added and the reaction

mixture was stirred for 15 h at room temperature. The solution was extracted with ethylacetate (3 × 20 mL) and the combined organic phases were washed with lithium chloride solution (3 × 20 mL, 5%). After being dried over sodium sulfate, the solvent was removed under reduced pressure. The residue was purified by column chromatography (pentane/EtOAc 2/1) to give the product as a light-yellow solid (0.84 g, 71%, 99% de, 99% ee).

R_f = 0.28 (pentane/EtOAc 2/1); mp 110–112 °C; $[\alpha]_D^{23}$ = −79.2 (*c* 1.0, $CHCl_3$). ^1H NMR (400 MHz, $CDCl_3$) δ 7.76 (s, 1H), 6.49 (s, 1H), 5.98–6.10 (m, 1H), 5.53 (dd, 1H, *J* = 17.3 Hz, 1.5 Hz), 5.48 (d, 2H, *J* = 6.6 Hz), 5.34 (dd, 1H, *J* = 10.6 Hz, 1.5 Hz), 4.98 (d, 1H, *J* = 6.6 Hz), 4.58–4.60 (m, 2H), 2.34 (s, 3H) ppm; ^{13}C NMR (101 MHz, $CDCl_3$) δ 205.4, 161.4, 159.4, 135.5, 132.0, 121.1, 118.0, 96.1, 91.3, 76.2, 72.2, 69.9, 28.4 ppm; IR (ATR): $\tilde{\nu}$ = 3330, 3224, 3068, 2927, 2868, 2704, 2324, 2184, 2112, 2003, 1890, 1722, 1613, 1585, 1478, 1449, 1404, 1368, 1322, 1286, 1263, 1207, 1172, 1131, 1061, 1025, 997, 939, 895, 834, 734, 667 cm^{-1}; MS (EI, 70 eV): *m/z* (%) = 360.0 (M^+, 100), 303.0 (15), 258.9 (36), 233.1 (38), 219.1 (12), 191.1 (14), 175.1 (12), 150.1 (25), 121.1 (15), 63.3 (17); anal. calcd. for $C_{13}H_{13}IO_4$: C 43.35, H 3.64; found: C 43.69, H 3.34; chiral HPLC: t_R = 16.31 min, 18.66 min (column: Daicel AD.M, *n*-Heptan/EtOH = 9/1, 1.0 mL/min).

(2*S*,3*R*)-6-(Allyloxy)-2-(2-hydroxypropan-2-yl)-5-iodo-2,3-dihydrobenzofuran-3-ol (15)

A $LaCl_3(LiCl)_2$ solution (0.51 M in THF, 2.86 mL, 1.46 mmol, 1.05 equiv) was poured into dry THF (6 mL) under an argon atmosphere. After the addition of compound **14** (500 mg, 1.39 mmol, 1.0 equiv), the solution was stirred for 1 h at room temperature. Then it was cooled down to 0 °C and methyl magnesium chloride (1.07 mL, 0.32 mmol, 2.3 equiv) was added slowly. After being stirred for 20 minutes, a solution

of sodium bicarbonate (5%, 1 mL), diethylether (2 mL) and Na-EDTA-solution (5%, 2 mL) were added. The aqueous phase was extracted with diethylether (2 × 5 mL) and the combined organic phases were dried over sodium sulfate. The solvent was removed under reduced pressure and the residue was purified by column chromatography (pentane/diethylether 6/4) to give the product as a colorless oil (453 mg, 87%).

R_f = 0.30 (pentane/diethylether 4/6); $[\alpha]_D^{23}$ = 36.3 (*c* 1.0, $CHCl_3$). ^{1}H NMR (400 MHz, $CDCl_3$) δ 7.70 (s, 1H), 6.42 (s, 1H), 5.96–6.07 (m, 1H), 5.50 (dd, 1H, *J* = 1.7 Hz, 17.3 Hz), 5.30 (dd, 1H, *J* = 1.7 Hz, 10.6 Hz), 5.22 (d, 1H, *J* = 6.2 Hz), 4.54 (d, 2H, *J* = 5.0 Hz), 4.21 (d, 1H, *J* = 6.2 Hz), 1.52 (s, 3H), 1.46 (s, 3H) ppm; ^{13}C NMR (101 MHz, $CDCl_3$) δ 161.2, 158.8, 135.1, 132.2, 124.3, 117.8, 96.0, 90.4, 75.5, 72.6, 72.3, 69.8, 28.2, 25.7 ppm; IR (ATR): $\tilde{\nu}$ = 3209, 2983, 2933, 2429, 2052, 1984, 1612, 1584, 1471, 1414, 1372, 1330, 1283, 1144, 1058, 1012, 933, 895, 864, 817, 790, 729, 668 cm^{-1}; MS (EI, 70 eV): *m/z* (%) = 376.0 (M^+, 100), 300.0 (72), 258.9 (78), 173.2 (23), 145.2 (13), 59.3 (76); anal. calcd. for $C_{14}H_{17}IO_4$: C 44.70, H 4.55; found: C 44.42, H 4.48.

(4a*S*,9b*R*)-7-(Allyloxy)-8-iodo-2,2,4,4-tetramethyl-4a,9b-dihydro-4*H*-[1,3]dioxino[5,4-b]benzofuran (16)

Compound **15** (250 mg, 0.67 mmol, 1.0 equiv), dimethoxypropane (693 mg, 6.66 mmol, 10.0 equiv), and pyridinium *p*-toluenesulfonate (11.0 mg, 0.07 mmol, 0.1 equiv) were stirred in acetone (10 mL, 0.07 M) at 50 °C for 24 h. Potassium carbonate (20.0 mg, 0.14 mmol, 0.2 equiv) was added, the solvent was removed under reduced pressure, and the crude product was purified by column chromatography (pentane/diethylether 20/1) to give the product as a colorless oil (183 mg, 66%).

R_f = 0.35 (pentane/diethylether 9/1); $[\alpha]_D^{23}$ = 37.1 (*c* 1.7, $CHCl_3$); ^{1}H NMR (400 MHz, $CDCl_3$) δ 7.70 (s, 1H), 6.36 (s, 1H), 5.96–6.08 (m, 1H), 5.50 (dd, 1H, *J* = 1.5 Hz, 17.3 Hz), 5.42 (d, 1H, *J* = 7.5 Hz), 5.30 (dd, 1H, *J* = 1.5 Hz, 10.6 Hz), 4.54 (d, 2H, *J* = 4.6 Hz) 4.46 (d, 1H, *J* = 4.6 Hz), 1.42 (s, 3H), 1.37 (s, 3H), 1.36 (s, 3H), 1.33 (s, 3H) ppm; ^{13}C NMR (101 MHz, $CDCl_3$) δ 162.2, 159.0, 135.8, 132.2, 128.5, 120.4, 117.8, 99.7, 95.4, 86.2, 75.0, 72.4, 70.5, 69.8, 29.9, 26.5, 23.6 ppm; IR (ATR): $\tilde{\nu}$ = 3919, 3555, 3367, 3085, 2983, 2928, 2563, 2431, 2353, 1861, 1779, 1594, 1470, 1418, 1375, 1327, 1287, 1228, 1186, 1142, 1069, 1017, 932, 889, 826, 729, 674, 613, 571, 482 cm^{-1}; MS (EI, 70 eV): *m/z* (%) = 416.1 (M^+, 100), 341.1 (33), 259.0 (44), 113.3 (30), 59.5 (18); HRMS-ESI: *m/z* calcd for $C_{17}H_{21}IO_4Na$ [M + Na^+]: 439.0377; found: 439.0377.

(4aS,9b*R*)-8-Iodo-2,2,4,4-tetramethyl-4a,9b-dihydro-4*H*-[1,3]dioxino[5,4-b]benzofuran-7-ol (17)

Under an argon atmosphere compound **16** (100 mg, 0.240 mmol, 1.0 equiv) was dissolved in methanol (2.4 mL, 0.1 M). Tetrakis(triphenylphosphine)palladium(0) (2.78 mg, 0.002 mmol, 0.01 equiv) was added and the solution was stirred for 5 minutes. After addition of potassium carbonate (100 mg, 0.720 mmol, 3.0 equiv), the reaction mixture was stirred for 48 h. The solvent was removed under reduced pressure, water (1 mL) and DCM (1 mL) were added, and the aqueous phase was extracted with DCM (3 × 1 mL). The combined organic phases were dried over sodium sulfate. After removal of the solvent under reduced pressure the residue was purified by column chromatography (pentane/diethylether 8/2) to give the product as a yellow oil (68 mg, 76%).

R_f = 0.42 (pentane/diethylether 1/1); $[\alpha]_D^{23}$ = 57.7 (*c* 1.7, $CHCl_3$); ^{1}H NMR (600 MHz, $CDCl_3$) δ 7.60 (s, 1H), 6.53 (s, 1H), 5.43 (d, 1H, *J* = 7.4 Hz), 4.49 (d, 1H, *J* = 7.4 Hz),

1.44 (s, 3H), 1.43 (s, 3H), 1.39 (s, 3H), 1.36 (s, 3H) ppm; ^{13}C NMR (151 MHz, $CDCl_3$) δ 162.7, 156.6, 134.5, 121.1, 99.8, 97.2, 86.3, 74.5, 72.5, 70.3, 29.9, 29.8, 26.4, 23.5 ppm; IR (ATR): $\tilde{\nu}$ = 3778, 3242, 2979, 2933, 2601, 2438, 2245, 1958, 1721, 1610, 1475, 1440, 1377, 1291, 1227, 1187, 1140, 1074, 1015, 915, 838, 733, 651, 565 cm^{-1}; MS (EI, 70 eV): *m/z* (%) = 375.9 (M^+, 43), 301.0 (34), 259.9 (100), 134.1 (16), 59.2 (20); anal. calcd. for $C_{14}H_{17}IO_4$: C 44.70, H 4.55; found: C 44.63, H 4.80.

tert-Butyl(((4a_S_,9b_R_)-8-iodo-2,2,4,4-tetramethyl-4a,9b-dihydro-4_H_-[1,3]dioxino[5,4-b]benzofuran-7-yl)oxy)dimethylsilane (18)

Tert-butylchlorodimethylsilane (167 mg, 1.11 mmol, 3.0 equiv) and DBU (197 mg, 1.29 mmol, 4.0 equiv) were dissolved in DCM (0.74 mL, 0.50 M) at room temperature. Compound **17** (139 mg, 0.369 mmol, 1.0 equiv) was added and the solution was stirred for 2 h. After being washed with saturated ammonium chloride solution (0.5 mL) and sodium bicarbonate solution (5%, 0.5 mL) the combined organic phases were dried over sodium sulfate and the solvent was removed under reduced pressure. The residue was purified by column chromatography to give the product as a colorless solid (156 mg, 91%).

R_f = 0.56 (pentane/diethylether 9/1); mp 91–94 °C; $[\alpha]_D^{23}$ = 45.6 (*c* 1.0, $CHCl_3$). ^{1}H NMR (600 MHz, $CDCl_3$) δ 7.69 (s, 1H), 6.37 (s, 1H), 5.42 (d, 1H, *J* = 7.4 Hz), 4.46 (d, 1H, *J* = 7.4 Hz), 1.44 (s, 3H), 1.39 (s, 3H), 1.38 (s, 3H), 1.36 (s, 3H), 1.05 (s, 9H), 0.28 (s, 6H) ppm; ^{13}C NMR (151 MHz, $CDCl_3$) δ 162.0, 157.0, 135.7, 121.0, 100.7, 99.8, 86.2, 79.3, 72.5, 70.4, 29.9, 29.8, 26.4, 25.9 (3C), 23.6, 18.4, −4.1 (2C) ppm; IR (ATR): $\tilde{\nu}$ = 3192, 2932, 2860, 2296, 2182, 2095, 1934, 1726, 1609, 1579, 1470, 1412, 1372, 1333, 1299, 1251, 1226, 1184, 1141, 1069, 1006, 976, 939, 873, 837, 779, 720, 673 cm^{-1}; MS (EI, 70 eV): *m/z* (%) = 490.2 (M^+, 23), 433.1 (100), 317.0

(15), 231.1 (18), 190.1 (34); HRMS-ESI: *m*/*z* calcd for $C_{20}H_{31}IO_4SiNa$ [M + Na$^+$]: 513.0929; found: 513.0920.

Ethyl 3-((4a*S*,9b*R*)-7-((*tert*-butyldimethylsilyl)oxy)-2,2,4,4-tetramethyl-4a,9b-dihydro-4*H*-[1,3]dioxino[5,4-b]benzofuran-8-yl)propiolate (19)

Under an argon atmosphere compound **18** (96.0 mg, 0.196 mmol, 1.0 equiv) and 3,3,3-triethoxy-1-propyne (mixture with ethoxyethyne 50 wt%, 83.0 mg, 0.242 mmol, 1.24 equiv) were dissolved in THF/triethylamine (1/1, 796 mL, 0.25 M) and degased. CuI (3.73 mg, 0.020 mmol, 0.10 equiv) and tetrakis(triphenylphosphine)palladium (11.3 mg, 0.010 mmol, 0.05 equiv) were added and the mixture was stirred for 30 h at 70 °C. The solution was washed with saturated ammonium chloride solution (1 mL), and the organic phase was dried over sodium sulfate. After removal of the solvent under reduced pressure the residue was dissolved in THF (2 mL), PPTA (2 mg, 0.012 mmol, 0.6 equiv) was added and the solution was stirred for 0.5 h. Afterwards it was washed with sodium bicarbonate solution (5%, 1 mL), the organic phase was dried over sodium sulfate and the solvent was removed under reduced pressure. The crude product was purified by column chromatography (pentane/diethylether 20/1) to give a yellow oil (72.0 mg, 80%).

R_f = 0.39 (pentane/diethylether 9/1); $[\alpha]_D^{23}$ = 16.0 (*c* 0.8, $CHCl_3$); ^{1}H NMR (400 MHz, $CDCl_3$) δ 7.52 (s, 1H), 6.33 (s, 1H), 5.46 (d, 1H, *J* = 7.4 Hz), 4.50 (d, 1H, *J* = 7.4 Hz), 4.25 (q, 2H, *J* = 7.1 Hz), 1.44 (s, 3H), 1.41 (s, 3H), 1.37 (s, 3H), 1.33 (s, 3H), 1.31 (t, 3H, *J* = 7.1 Hz), 1.03 (s, 9H), 0.26 (s, 6H) ppm; ^{13}C NMR (101 MHz, $CDCl_3$) δ 163.8, 161.5, 132.3, 119.9, 105.2, 101.7, 99.8, 86.5, 84.9, 83.7, 76.4, 75.7, 72.3, 70.1, 61.5, 29.7, 26.6, 25.6 (3C), 23.7, 18.2, 14.1, −4.4 (2C) ppm; IR (ATR): $\tilde{\nu}$ = 3397, 3200, 2936, 2860, 2647, 2464, 2211, 1707, 1620, 1582, 1484, 1443, 1370, 1308, 1231, 1115, 1071, 1007, 977, 940, 882, 844, 785, 754, 710, 673, 589, 540, 490, 456 cm^{-1};

MS (EI, 70 eV): *m/z* (%) = 460.2 (M^+, 13), 403.1 (100), 359.0 (22), 301.0 (11), 273.0 (10), 246.9 (14), 215.0 (12), 113.0 (10), 59.1 (11); anal. calcd. for $C_{25}H_{36}O_6$: C 65.19, H 7.88; found: C 65.34, H 7.70.

Ethyl 3-((4a*S*,9b*R*)-7-hydroxy-2,2,4,4-tetramethyl-4a,9b-dihydro-4*H*-[1,3]dioxino[5,4-b]benzofuran-8-yl)propiolate (20)

Compound **19** (179 mg, 0.389 mmol, 1.0 equiv) and tetrabutylammonium fluoride (1 M solution in THF, 0.777 mL, 0.777 mmol, 2.0 equiv) were dissolved in THF (1.94 mL, 0.20 M) and stirred for 5 minutes at room temperature. The solution was washed with saturated ammonium chloride solution (1 mL) and the organic phase was dried over sodium sulfate. After removal of the solvent under reduced pressure the product was separated by column chromatography (pentane/diethylether 1/1) to give a yellow oil (108 mg, 98%).

R_f = 0.33 (pentane/diethylether 1/1); $[\alpha]_D^{23}$ = 23.1 (*c* 0.8, $CHCl_3$). ^{1}H NMR (400 MHz, $CDCl_3$) δ 7.48 (s, 1H), 6.40 (s, 1H), 5.40 (d, 1H *J* = 7.3 Hz), 4.49 (d, 1H, *J* = 7.3 Hz), 4.28 (q, 2H, *J* = 7.2 Hz), 1.44 (s, 3H), 1.40 (s, 3H), 1.36 (s, 3H), 1.34 (s, 3H), 0.90 (t, 3H, *J* = 7.2 Hz) ppm; ^{13}C NMR (101 MHz, $CDCl_3$) δ 164.8, 131.5, 125.5, 119.0, 99.8, 99.2, 97.8, 86.5, 72.4, 70.0, 62.0, 53.0, 30.3, 29.8, 29.8, 26.5, 23.6, 20.6, 14.1 ppm; IR (ATR): $\tilde{\nu}$ = 3242, 2982, 2935, 2193, 1700, 1656, 1616, 1495, 1454, 1373, 1272, 1211, 1185, 1107, 1068, 999, 970, 938, 892, 836, 750, 708, 667 cm^{-1}; MS (EI, 70 eV): *m/z* (%) = 346.2 (M^+, 19), 271.1 (16), 230.2 (21), 184.1 (100), 133.2 (24), 59.4 (30); HRMS-ESI: *m/z* calcd for $C_{19}H_{23}O_6$ [M + H^+]: 347.1489; found: 347.1489.

(4a*S*,11b*R*)-2,2,4,4-Tetramethyl-4,4a-dihydro-[1,3]dioxino[4',5':4,5]furo[3,2-g]chromen-8(11b*H*)-one (21)

Compound **20** (50.0 mg, 0.144 mmol, 1.0 equiv) was dissolved in ethyl acetate (2.1 mL, 0.07 M) and the solution was degased. Lindlar catalyst (5% Pd/$CaCO_3$, $Pb(OCOCH_3)_2$, 15 mg) was added under argon counter current, and the mixture was stirred for 15 minutes. Afterwards an atmosphere of hydrogen (1 atm) was generated and the mixture was stirred for a further 24 h at 30 °C. The catalyst was removed by filtration over celite. After removal of the solvent under reduced pressure, the product was isolated by column chromatography (pentane/diethylether 2/1) to give a colorless solid (40.0 mg, 92%).

R_f = 0.30 (pentane/diethylether 1/1); mp 150–152 °C; $[\alpha]_D^{23}$ = −40.0 (*c* 0.5, $CHCl_3$). ^1H NMR (400 MHz, $CDCl_3$) δ 7.63 (d, 1H, *J* = 9.5 Hz), 7.49 (s, 1H), 6.79 (s, 1H), 6.24 (d, 1H, *J* = 9.5 Hz), 5.54 (d, 1H, *J* = 7.4 Hz), 4.59 (d, 1H, *J* = 7.4 Hz), 1.48 (s, 3H), 1.43 (s, 3H), 1.38 (s, 6H) ppm; ^{13}C NMR (101 MHz, $CDCl_3$) δ 163.8, 161.1, 157.2, 143.8, 125.7, 124.2, 113.4, 112.9, 100.1, 98.7, 86.8, 72.6, 70.1, 30.0, 29.9, 26.5, 23.6 ppm; IR (ATR): $\tilde{\nu}$ = 3090, 2981, 2936, 2068, 1712, 1621, 1568, 1483, 1379, 1324, 1253, 1187, 1121, 1003, 906, 835, 730 cm^{-1}; MS (EI, 70 eV): *m/z* (%) = 302.3 (M^+, 32), 227.2 (34), 186.2 (100), 158.2 (23); HRMS-ESI: *m/z* calcd for $C_{17}H_{18}O_5Na$ [M + Na^+]: 325.1046; found: 325.1045.

(2*S*,3*R*)-3-Hydroxy-2-(2-hydroxypropan-2-yl)-2*H*-furo[3,2-g]chromen-7(3*H*)-one (1)

Compound **21** (21.0 mg, 0.069 mmol, 1.0 equiv), water (5.0 mg, 0.28 mmol, 4.0 equiv) and indium(III) chloride (46.1 mg, 0.21 mmol, 3.0 equiv) were dissolved in acetonitrile (1.04 mL, 0.07 M) and stirred at room temperature for 3 h. After the addition of water (2 mL) and DCM (2 mL), the aqueous phase was extracted with

DCM (3 x 1 mL) and dried over sodium sulfate. After removal of the solvent under reduced pressure, the product was isolated by column chromatography (pentane/diethylether 1/1, then ethylacetate) to give a colorless solid (17.0 mg, 93%). R_f = 0.50 (ethylacetate); mp 162–164°C (lit. [9] mp 164–166 °C); $[\alpha]_D^{23}$ = 40.2 (*c* 0.5, $CHCl_3$) (lit. [9] $[\alpha]_D^{23}$ = 22.4 (c = 0.25, $CHCl_3$)). ^1H NMR (600 MHz, $CDCl_3$) δ 7.63 (d, 1H, *J* = 9.5 Hz), 7.50 (s, 1H), 6.82 (s, 1H), 6.24 (d, 1H, *J* = 9.5 Hz), 5.37 (d, 1H, *J* = 6.1 Hz), 4.36 (d, 1H, *J* = 6.1 Hz), 1.59 (s, 3H), 1.54 (s, 3H) ppm; ^{13}C NMR (151 MHz, $CDCl_3$) δ 162.4, 161.0, 156.7, 143.7, 128.1, 124.8, 113.6, 112.9, 99.0, 90.5, 73.0, 71.8, 28.4, 25.3 ppm; IR (ATR): $\tilde{\nu}$ = 3434, 2921, 2853, 1712, 1579, 1547, 1462, 1384, 1112, 530 cm^{-1}; MS (EI, 70 eV): *m/z* (%) = 262.0 (M^+, 21), 186.0 (100), 158.0 (33), 59.2 (34); HRMS-ESI: *m/z* calcd for $C_{14}H_{15}O_5$ [M + H^+]: 263.0914; found: 263.0912. The ^1H and ^{13}C NMR data match with the literature data [9].
