# Supplementary material for: Asymmetric total synthesis of smyrindiol employing an organocatalytic aldol key step
Source: Beilstein J Org Chem. 2012 Jul 18;8:1112–7. doi: 10.3762/bjoc.8.123 (PMC3458728; doi:10.3762/bjoc.8.123)
Supplement: File 2 — NMR-spectra and chromatograms. [file Beilstein_J_Org_Chem-08-1112-s002.pdf]

Supporting Information File 2

for

Asymmetric total synthesis of smyrindiol employing an organocatalytic aldol key step

Dieter Enders*, Jeanne Fronert, Tom Bisschops and Florian Boeck

Address: Institute of Organic Chemistry, RWTH Aachen University, Landoltweg 1, 52074 Aachen, Germany

Email: Dieter Enders - enders@rwth-aachen.de

*Corresponding author

NMR-spectra and chromatograms

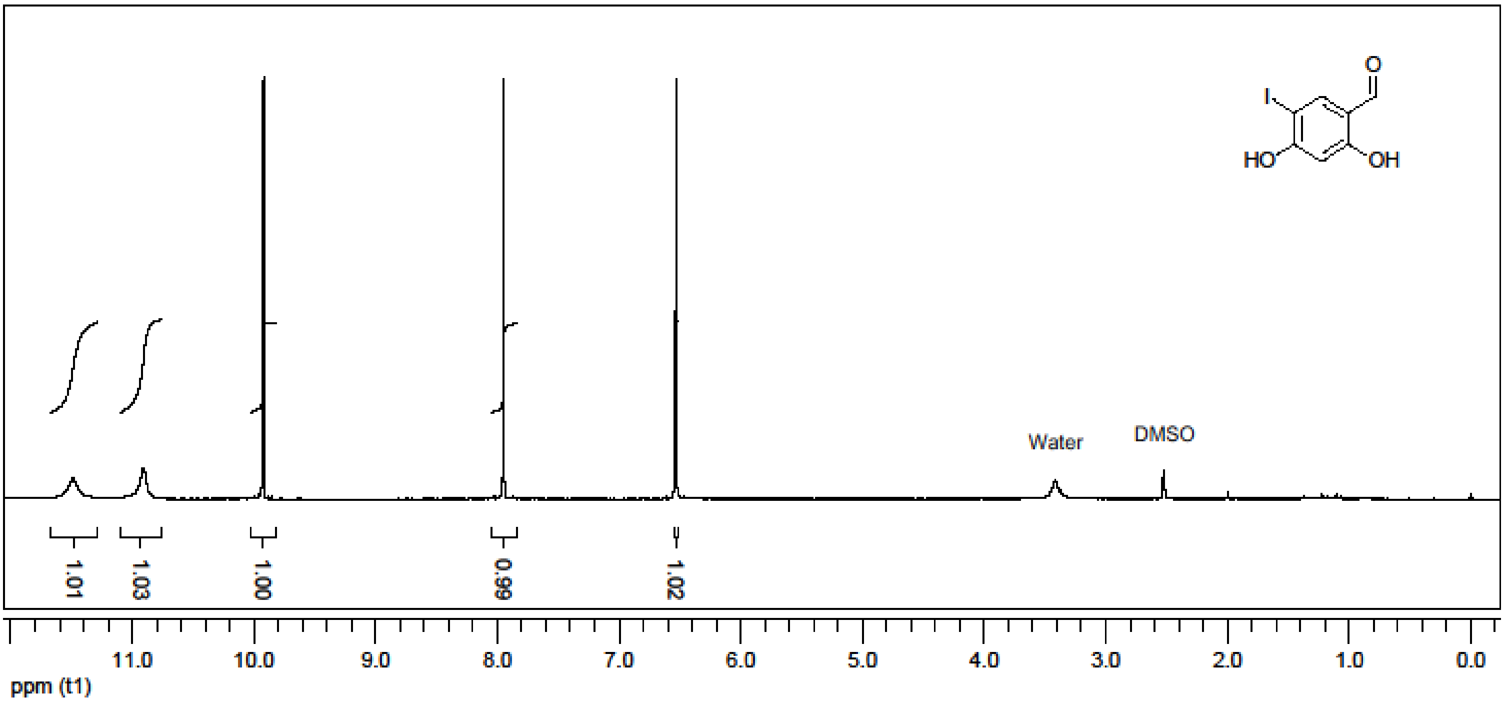
O
I
HO
OH
Water
DMSO
1.01
1.03
1.00
0.99
1.02
11.0
10.0
9.0
8.0
7.0
6.0
5.0
4.0
3.0
2.0
1.0
0.0
ppm (t1)

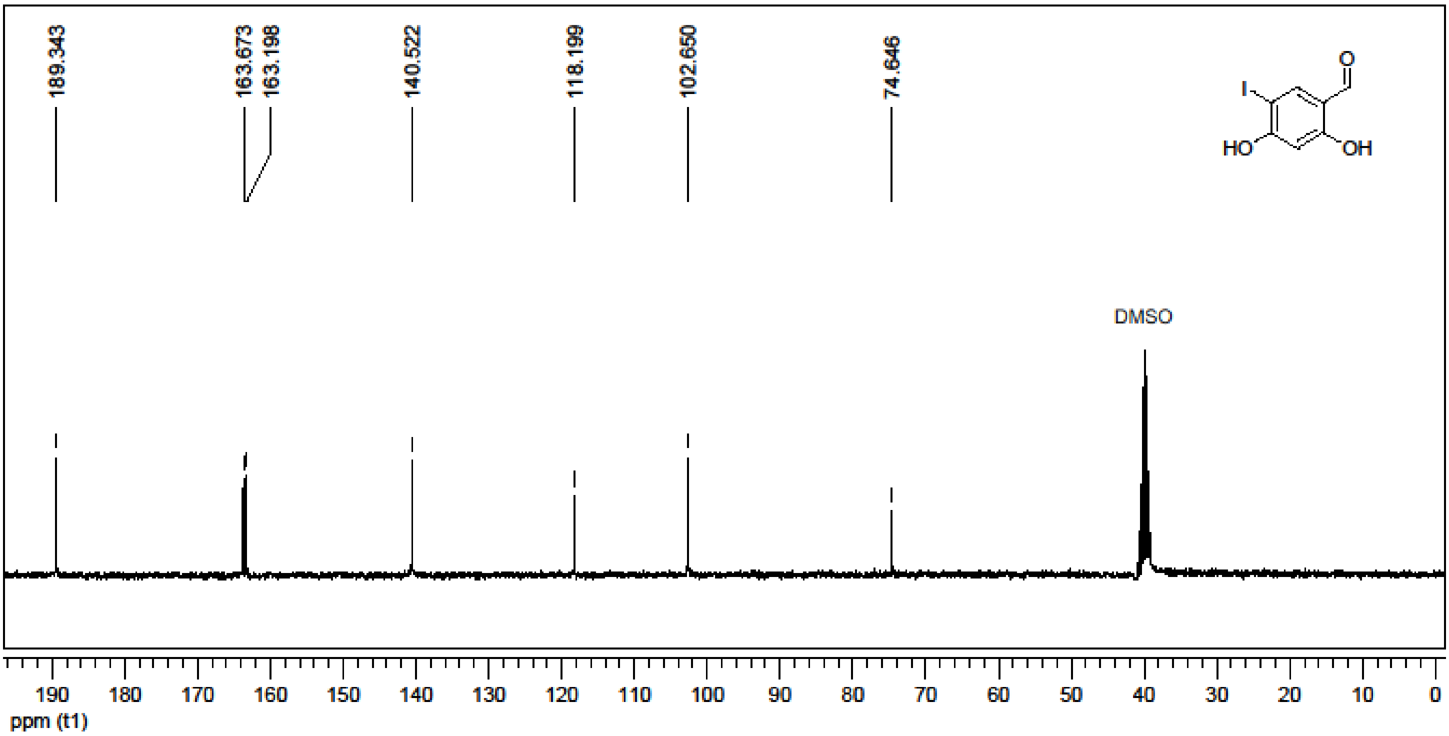
189.343
163.673
163.198
140.522
118.199
102.650
74.646
O
I
HO
OH
DMSO
190
180
170
160
150
140
130
120
110
100
90
80
70
60
50
40
30
20
10
0
ppm (t1)

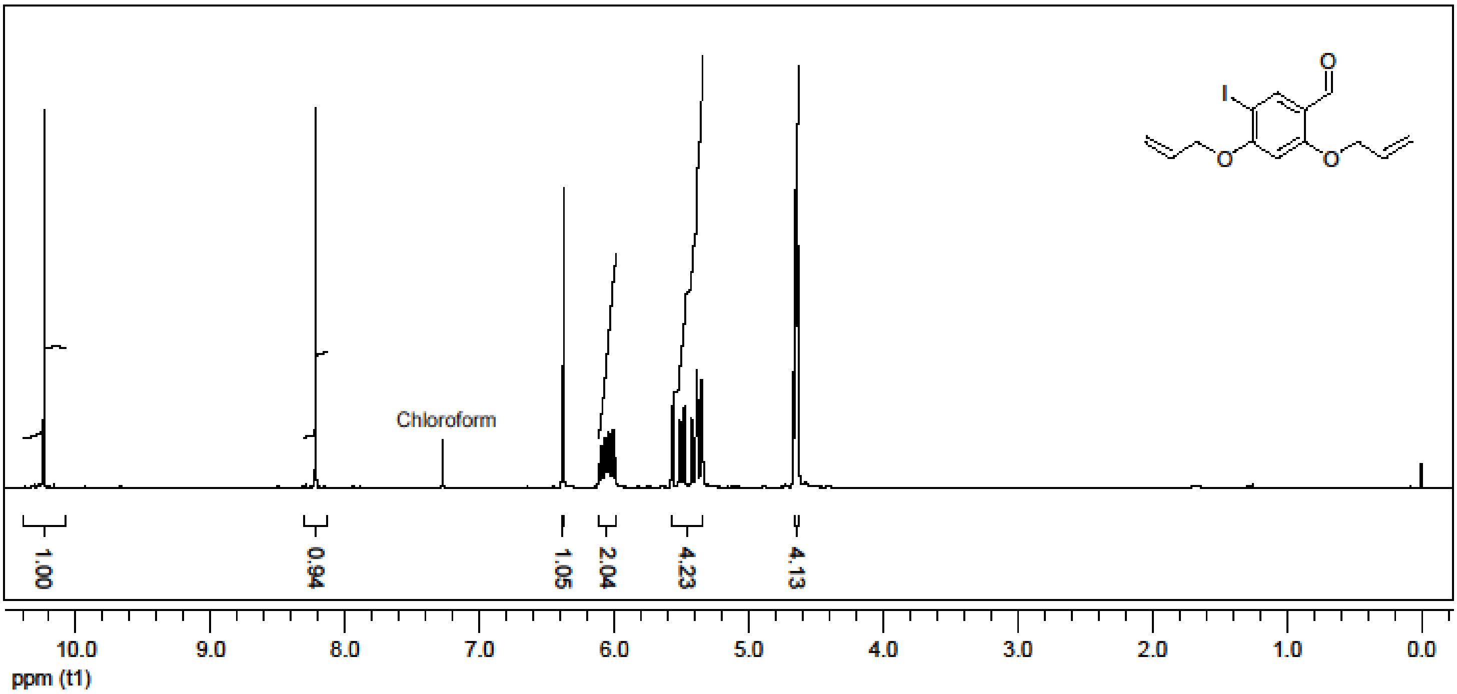
Chloroform
1.00
0.94
1.05
2.04
4.23
4.13
10.0
9.0
8.0
7.0
6.0
5.0
4.0
3.0
2.0
1.0
0.0
ppm (t1)
I
O
O
O

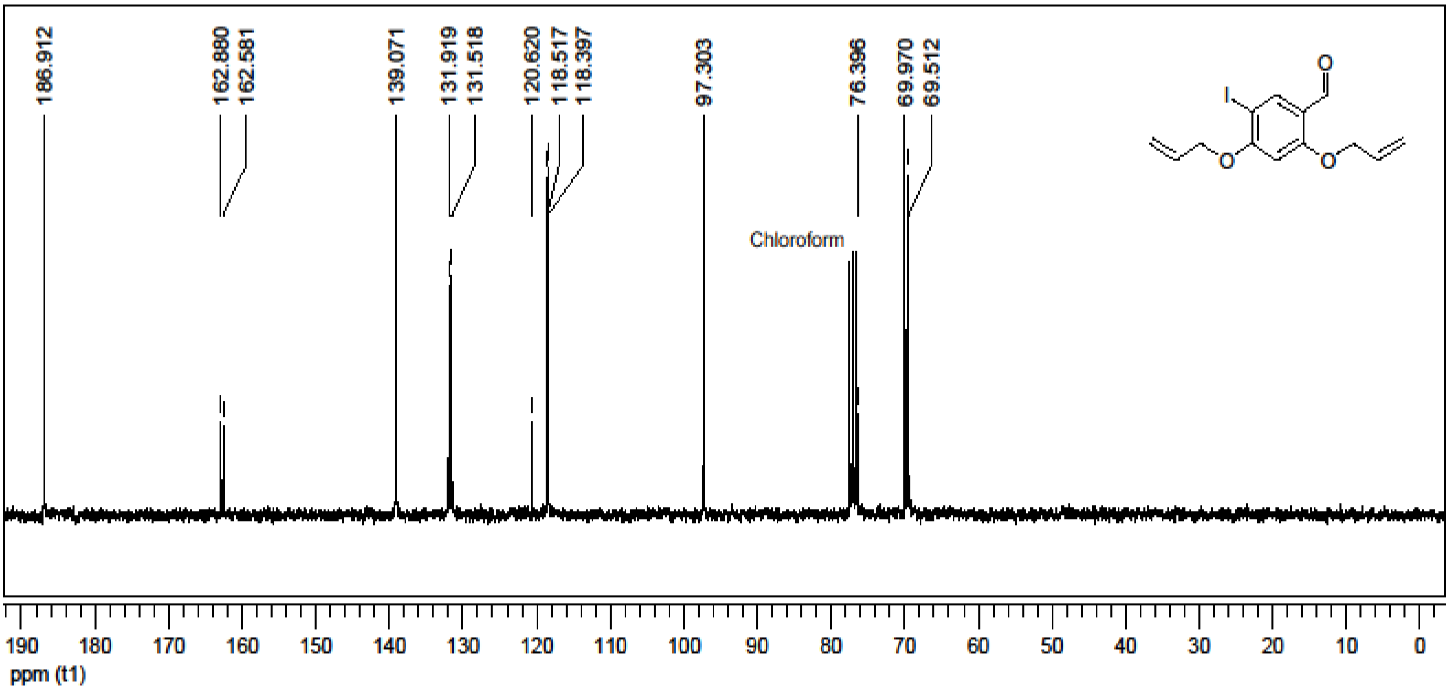
186.912
162.880
162.581
139.071
131.919
131.518
120.620
118.517
118.397
97.303
76.396
69.970
69.512
Chloroform
190
180
170
160
150
140
130
120
110
100
90
80
70
60
50
40
30
20
10
0
ppm (t1)
I
O
O
O

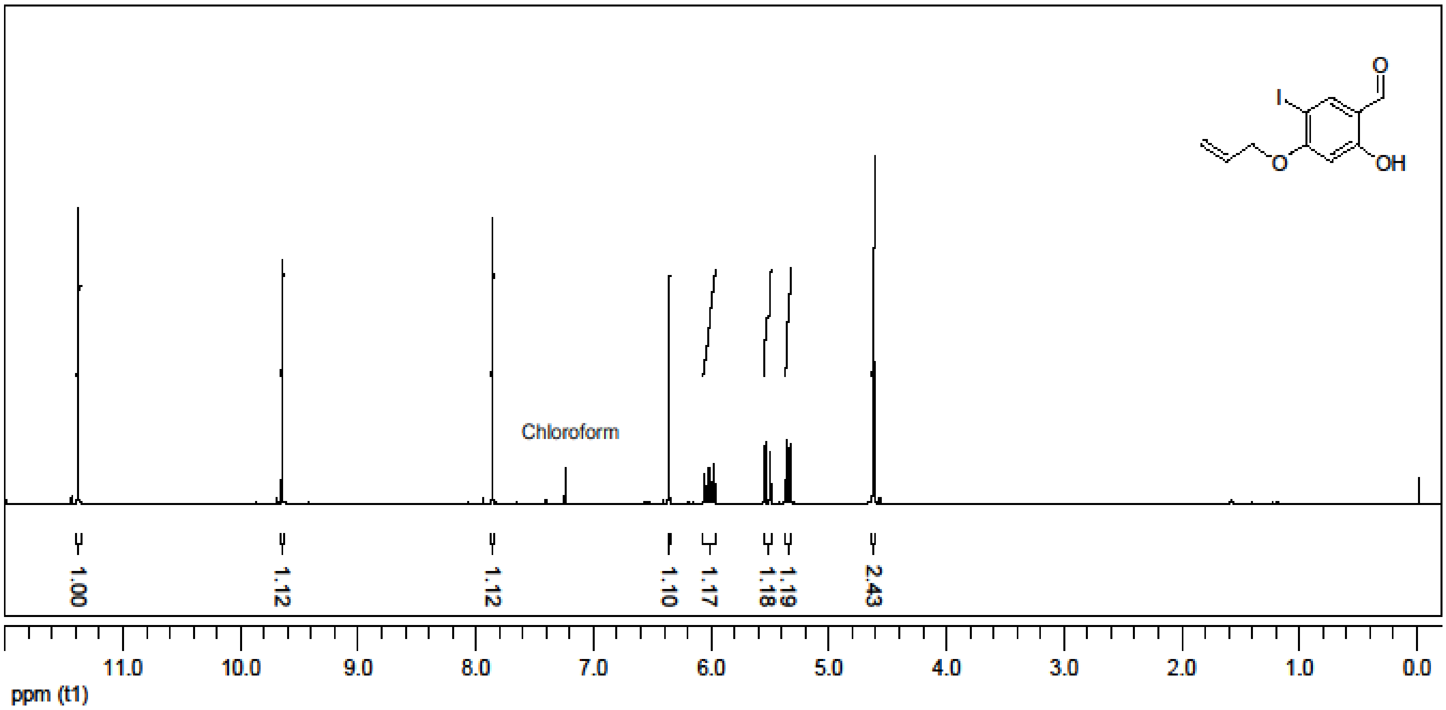
Chloroform
1.00
1.12
1.12
1.10
1.17
1.18
1.19
2.43
11.0
10.0
9.0
8.0
7.0
6.0
5.0
4.0
3.0
2.0
1.0
0.0
ppm (t1)
I
O
OH
O

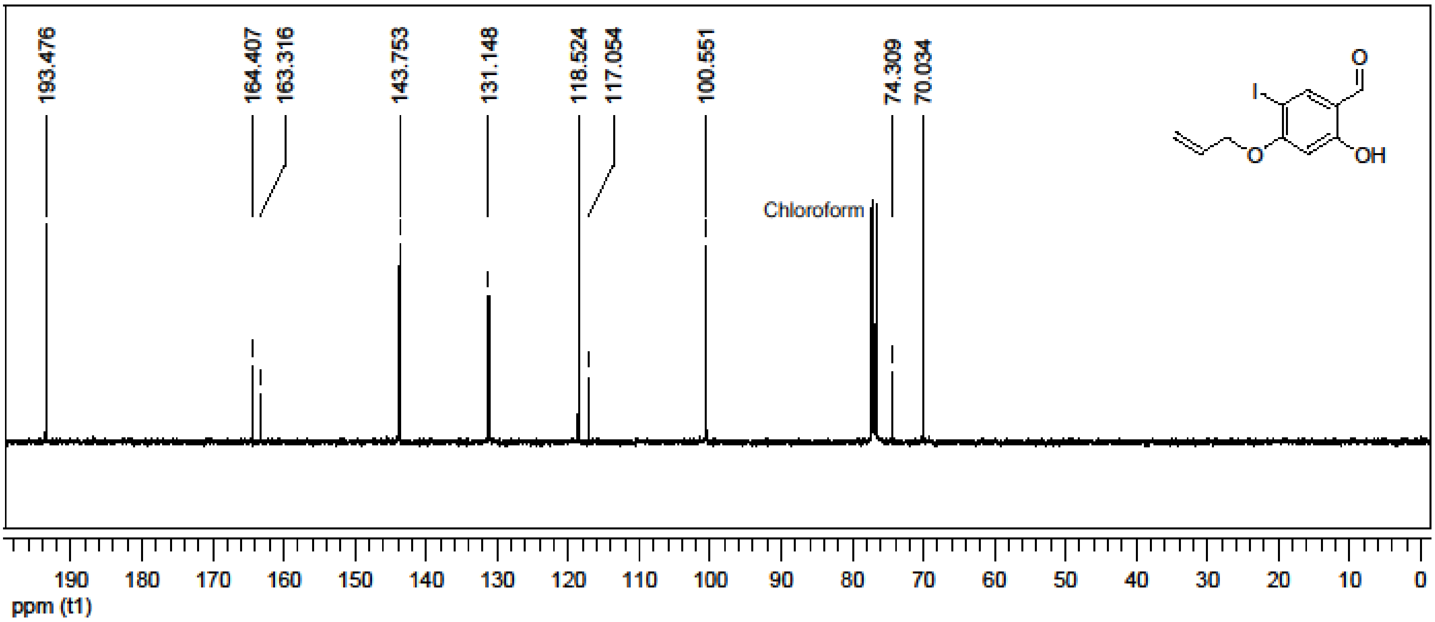
193.476
164.407
163.316
143.753
131.148
118.524
117.054
100.551
74.309
70.034
Chloroform
190
180
170
160
150
140
130
120
110
100
90
80
70
60
50
40
30
20
10
0
ppm (t1)
I
O
OH
O

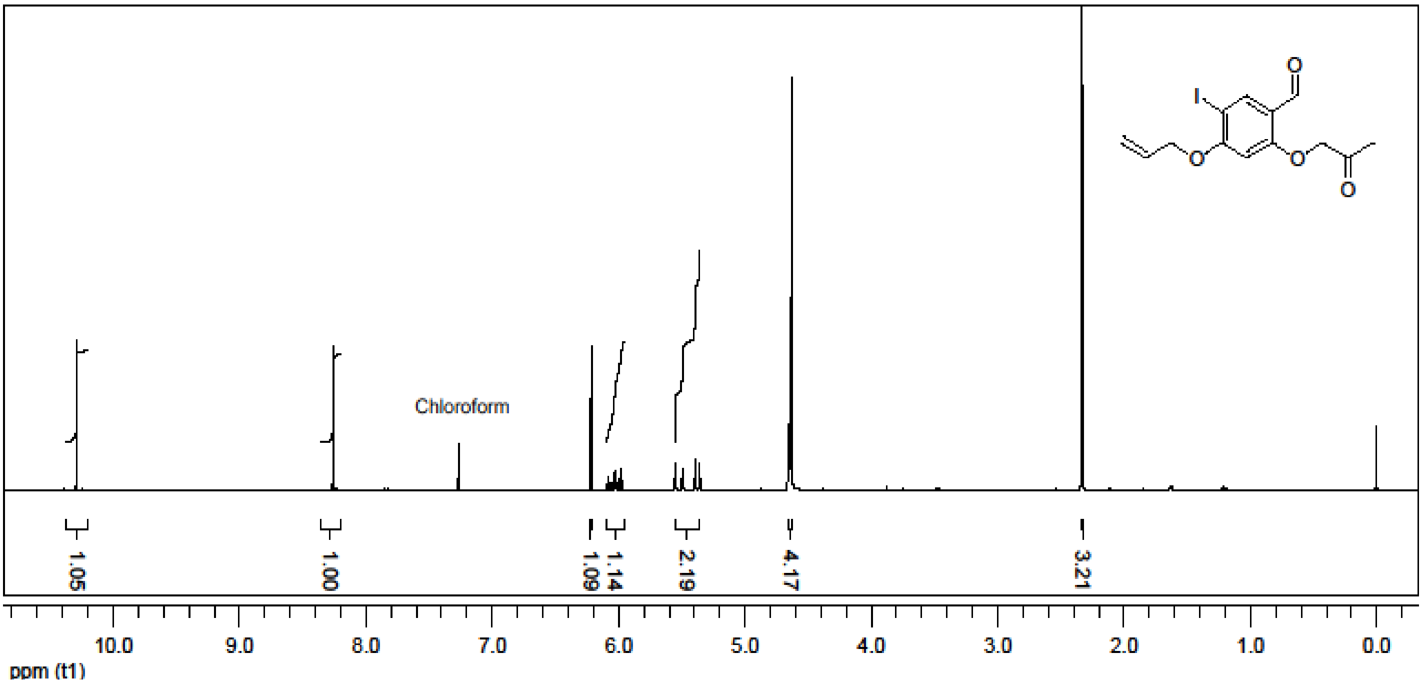
Chloroform
I
O
O
O
O
1.05
1.00
1.09
1.14
2.19
4.17
3.21
10.0
9.0
8.0
7.0
6.0
5.0
4.0
3.0
2.0
1.0
0.0
ppm (t1)

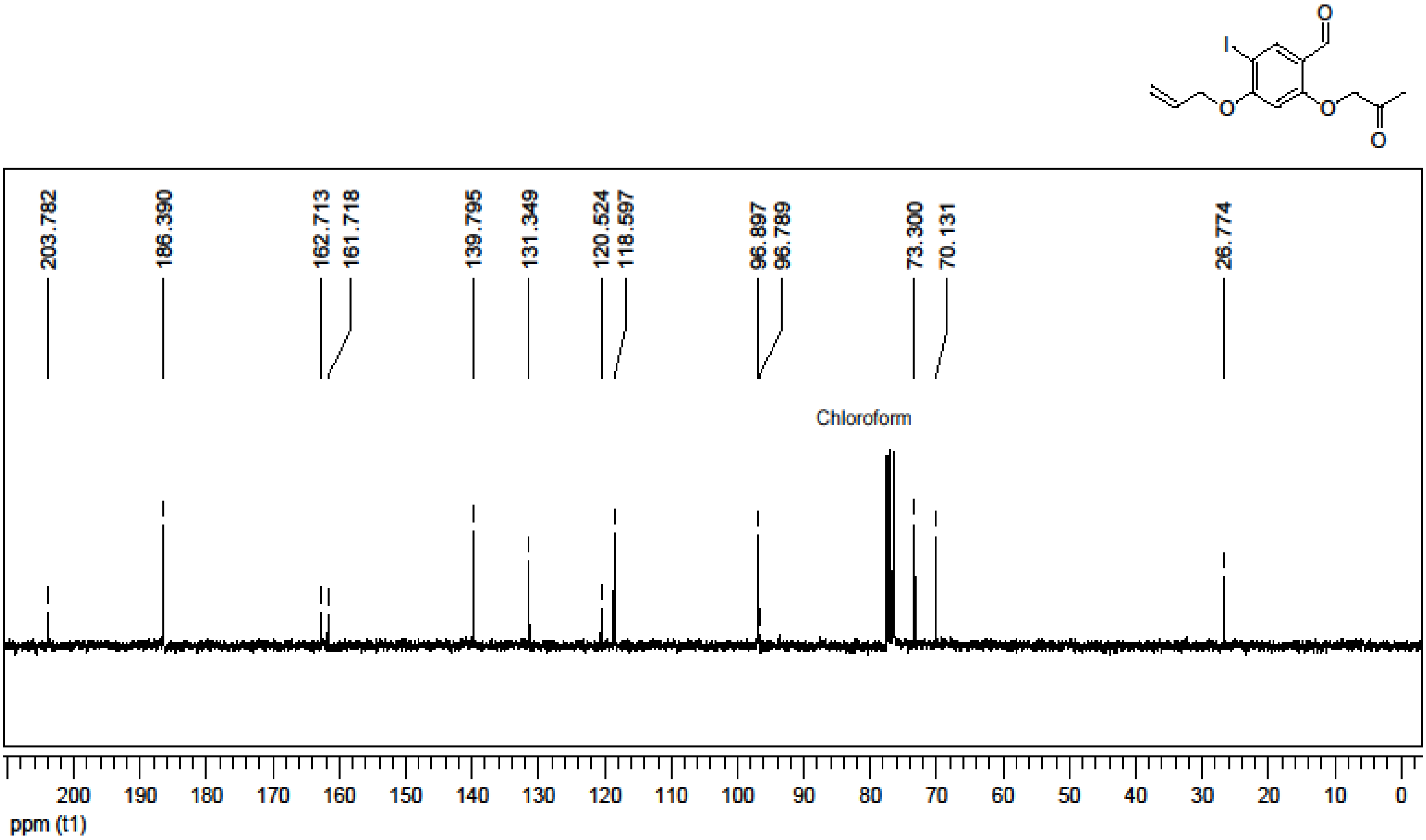
I
O
O
O
O
203.782
186.390
162.713
161.718
139.795
131.349
120.524
118.597
96.897
96.789
73.300
70.131
26.774
Chloroform
200
190
180
170
160
150
140
130
120
110
100
90
80
70
60
50
40
30
20
10
0
ppm (t1)

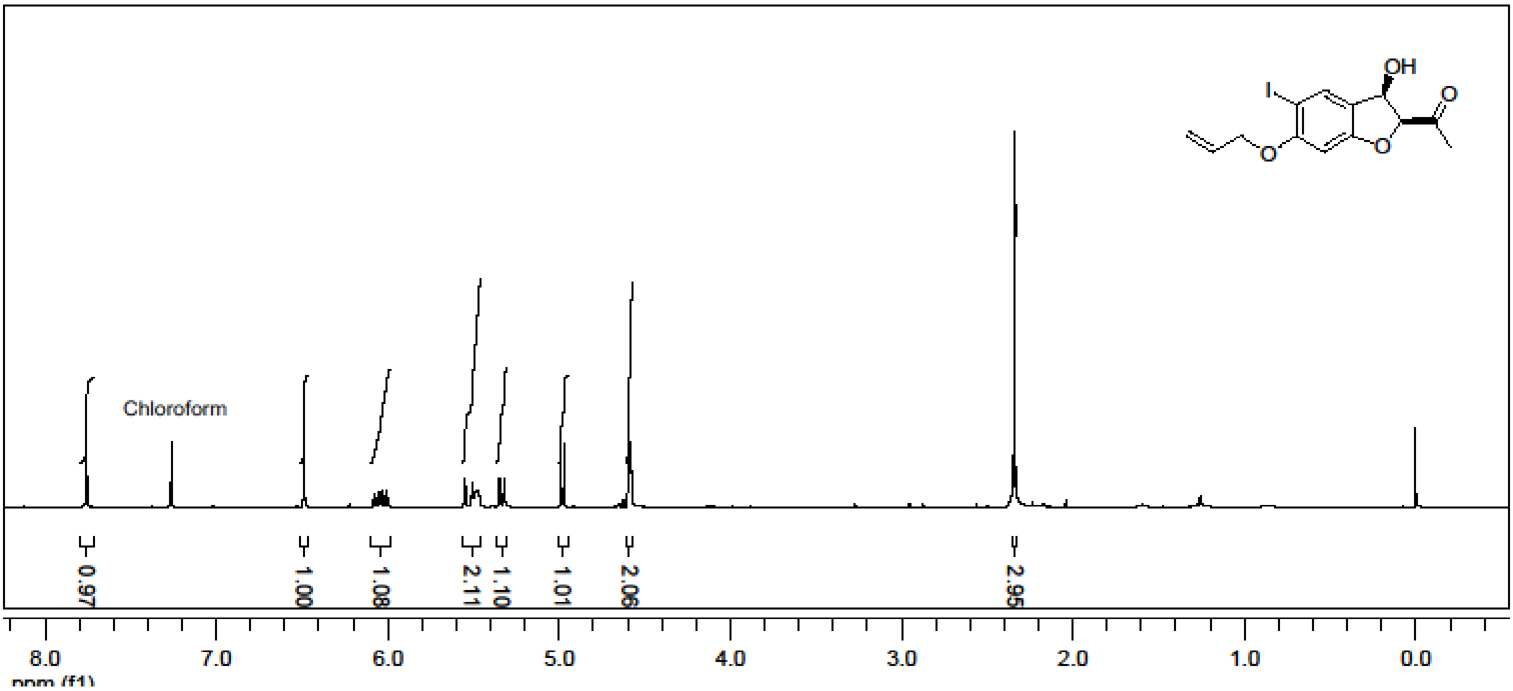
OH
I
O
O
O
Chloroform
0.97
1.00
1.08
2.11
1.10
1.01
2.06
2.95
8.0 7.0 6.0 5.0 4.0 3.0 2.0 1.0 0.0
ppm (f1)

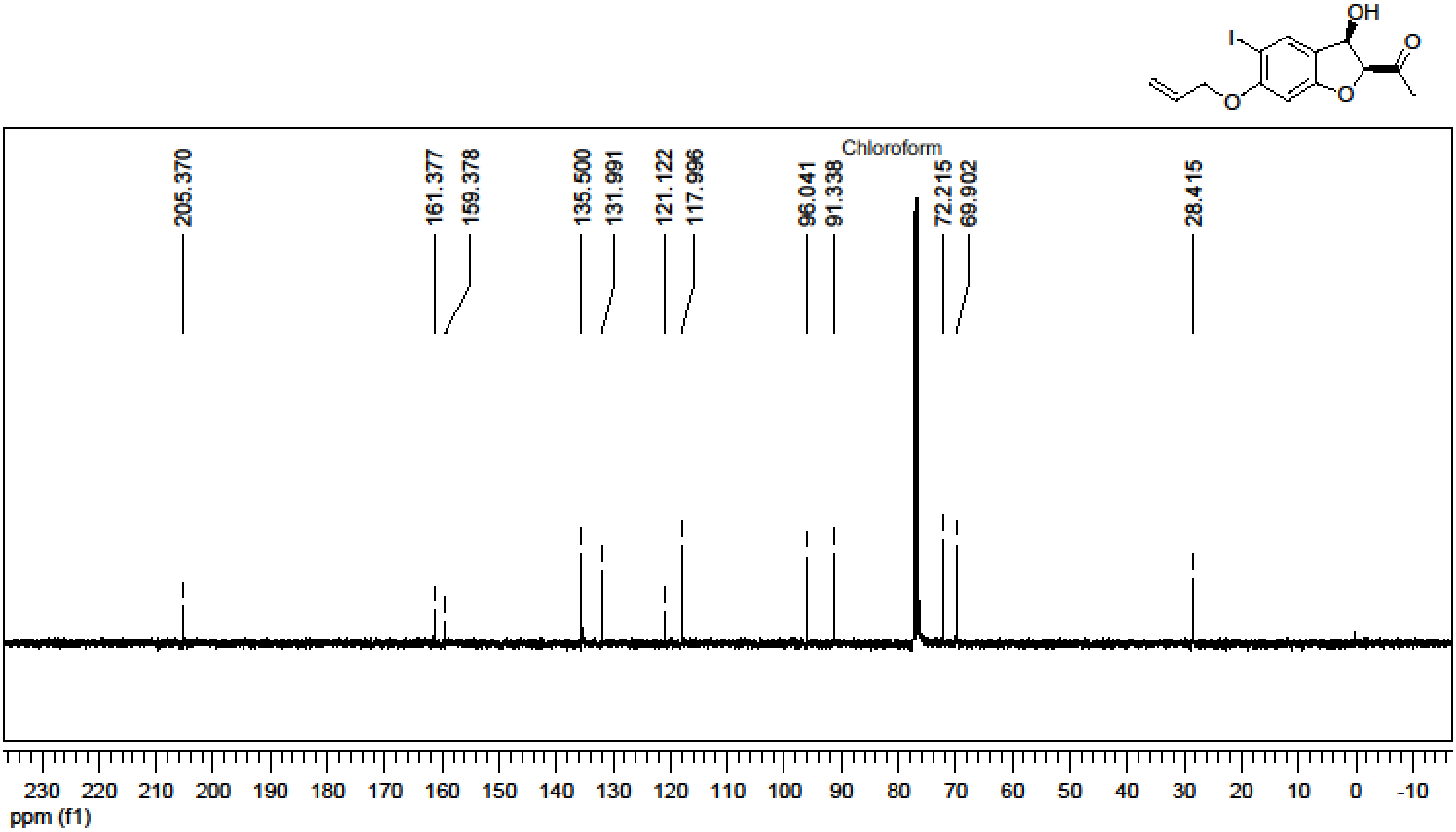
OH
I
O
O
O
Chloroform
205.370
161.377
159.378
135.500
131.991
121.122
117.996
96.041
91.338
72.215
69.902
28.415
230 220 210 200 190 180 170 160 150 140 130 120 110 100 90 80 70 60 50 40 30 20 10 0 -10
ppm (f1)

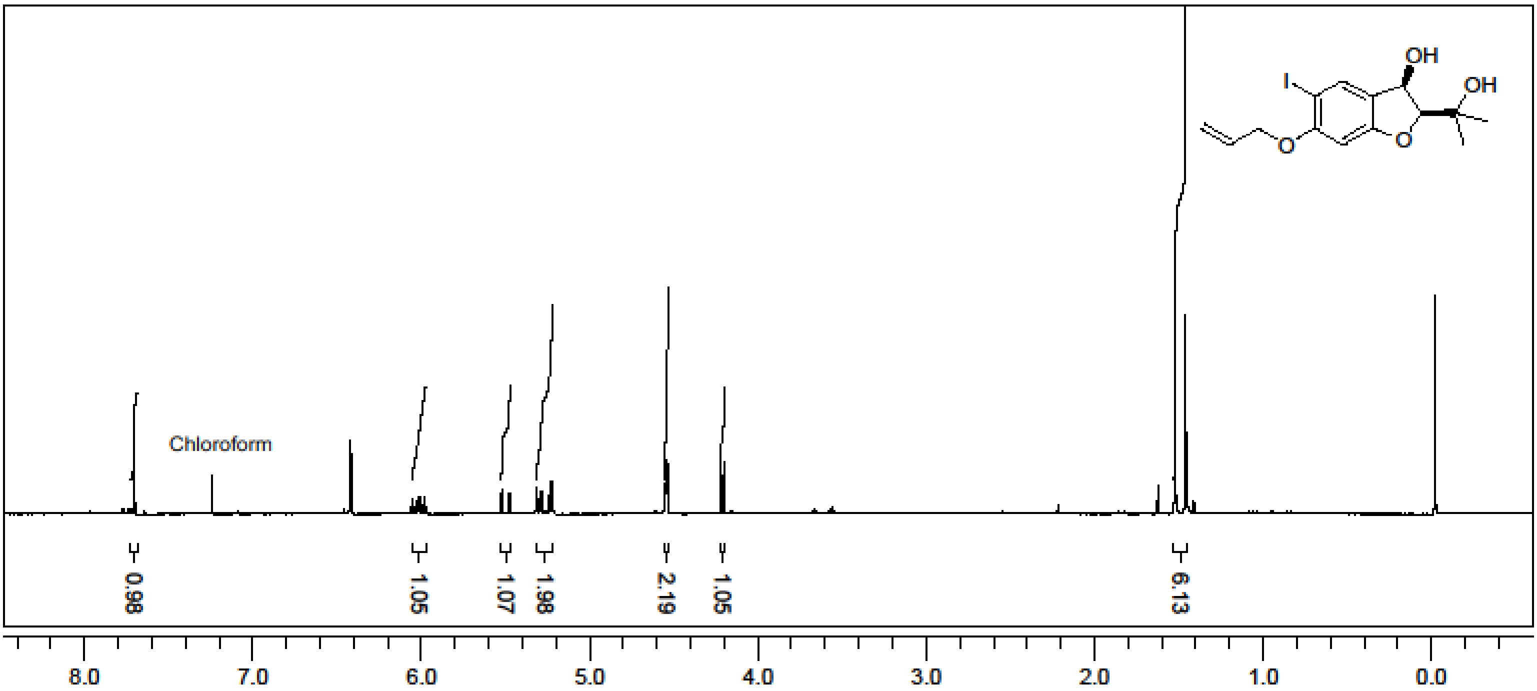
OH
I
OH
O
O
Chloroform
0.98
1.05
1.07
1.98
2.19
1.05
6.13
8.0
7.0
6.0
5.0
4.0
3.0
2.0
1.0
0.0

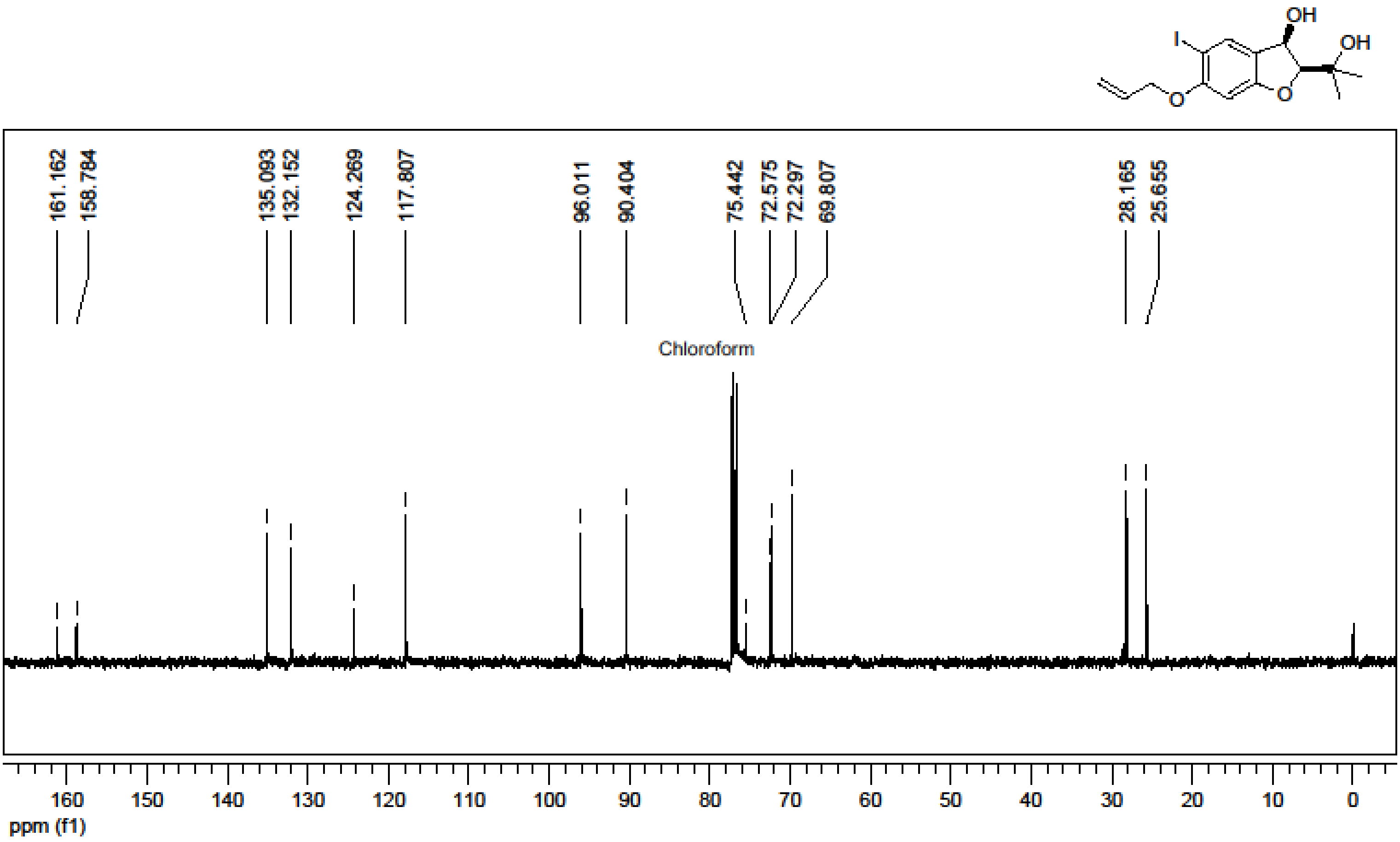
OH
I
OH
O
O
161.162
158.784
135.093
132.152
124.269
117.807
96.011
90.404
75.442
72.575
72.297
69.807
28.165
25.655
Chloroform
160
150
140
130
120
110
100
90
80
70
60
50
40
30
20
10
0
ppm (f1)

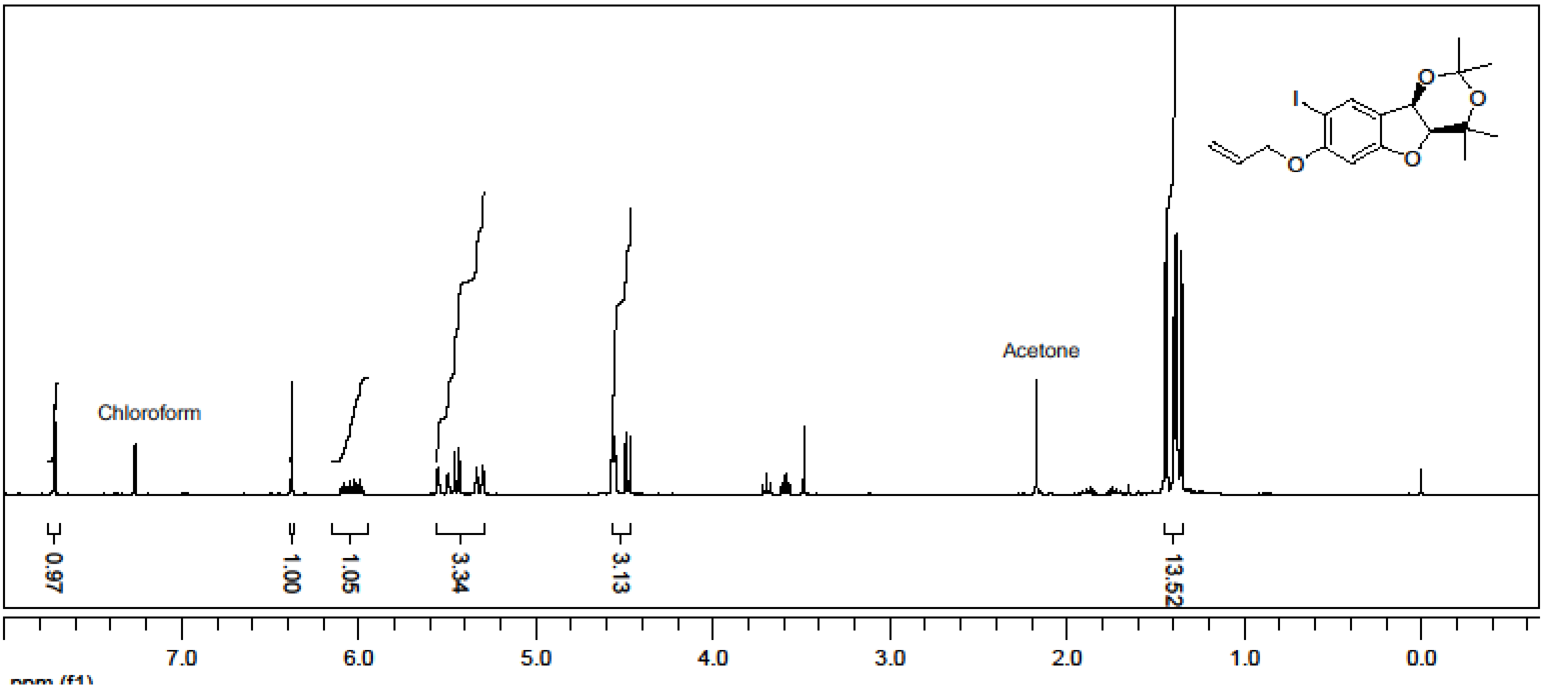
Chloroform
Acetone
I
O
0.97
1.00
1.05
3.34
3.13
13.52
7.0
6.0
5.0
4.0
3.0
2.0
1.0
0.0

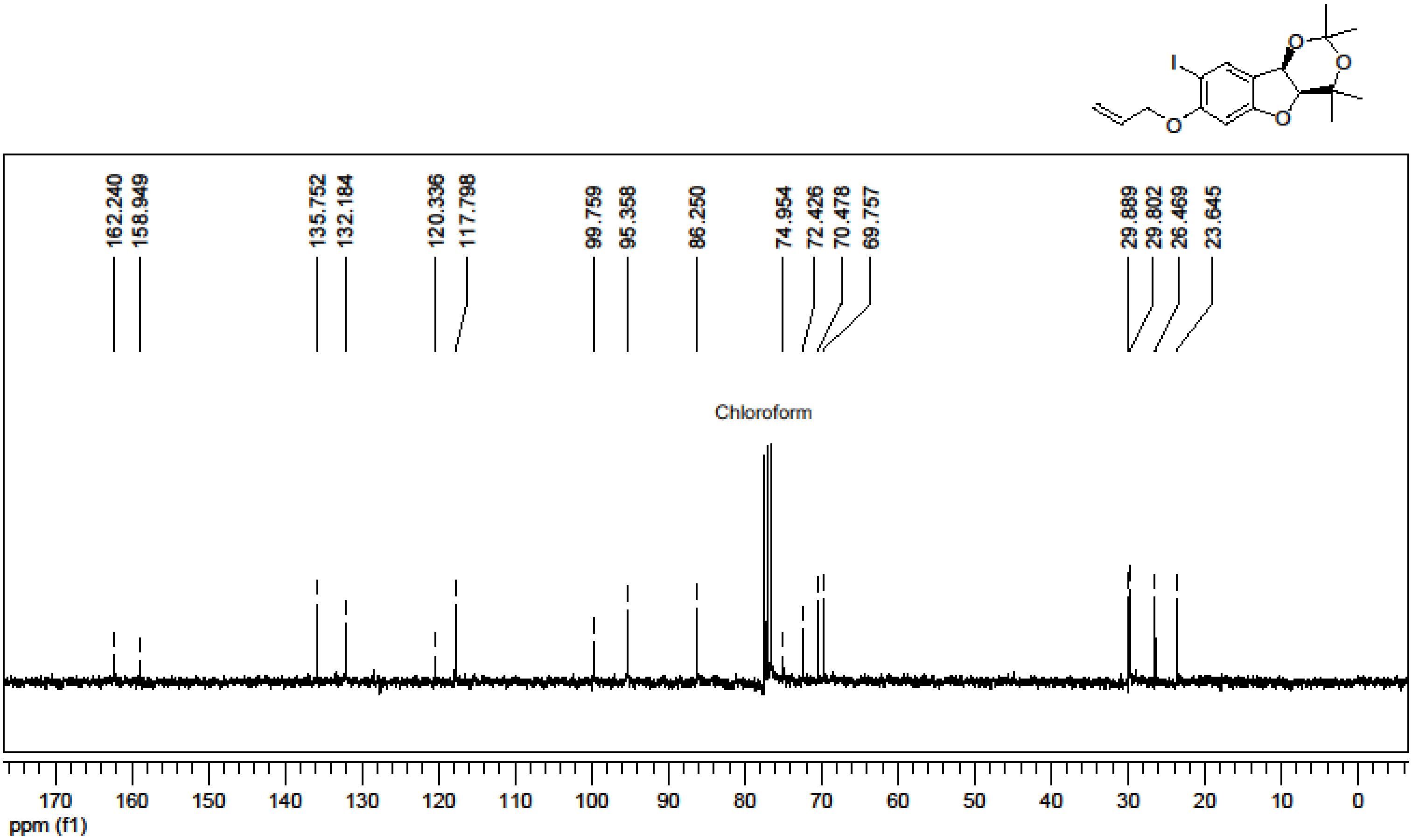
I
O
162.240
158.949
135.752
132.184
120.336
117.798
99.759
95.358
86.250
74.954
72.426
70.478
69.757
29.889
29.802
26.469
23.645
Chloroform
170
160
150
140
130
120
110
100
90
80
70
60
50
40
30
20
10
0
ppm (f1)

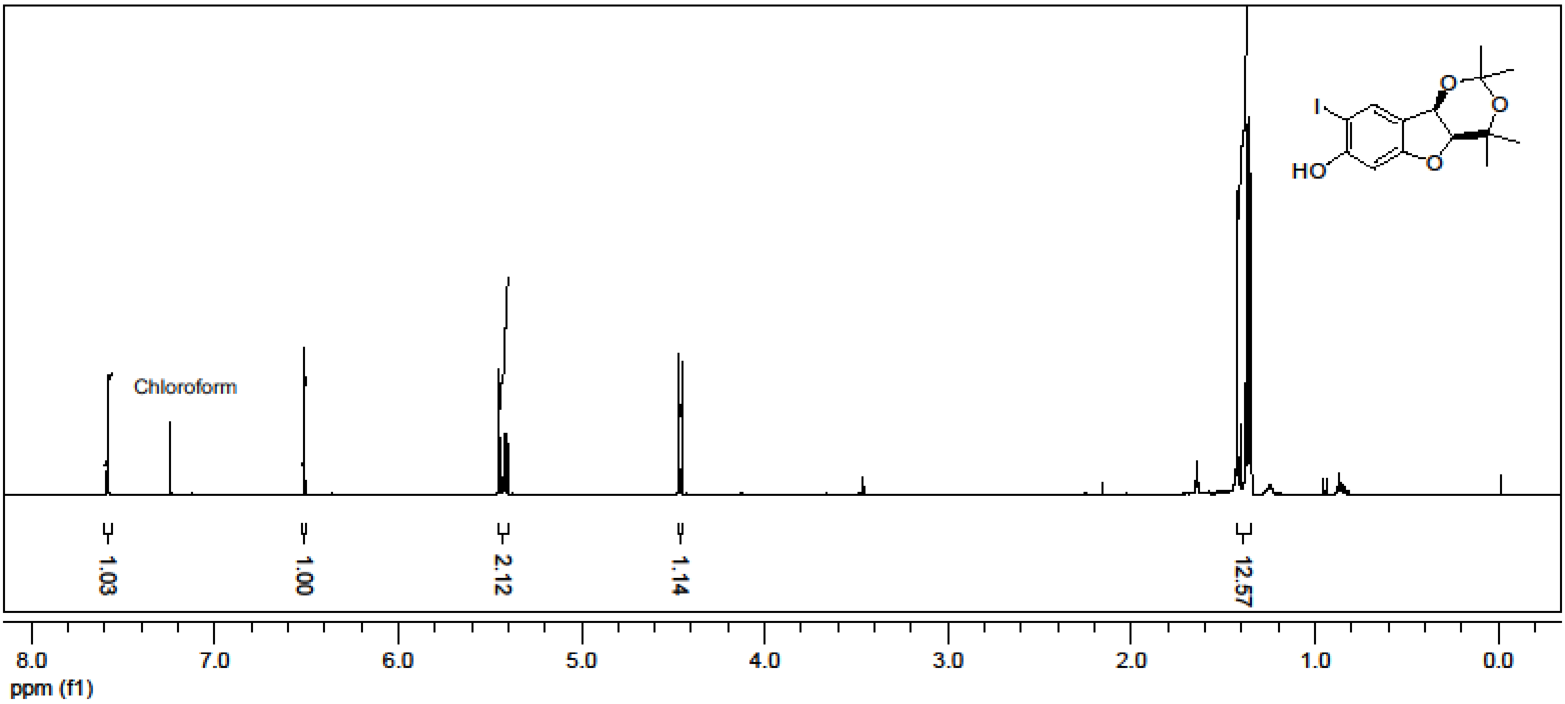
Chloroform
1.03
1.00
2.12
1.14
12.57
8.0
7.0
6.0
5.0
4.0
3.0
2.0
1.0
0.0
ppm (f1)
I
HO
O
O
O

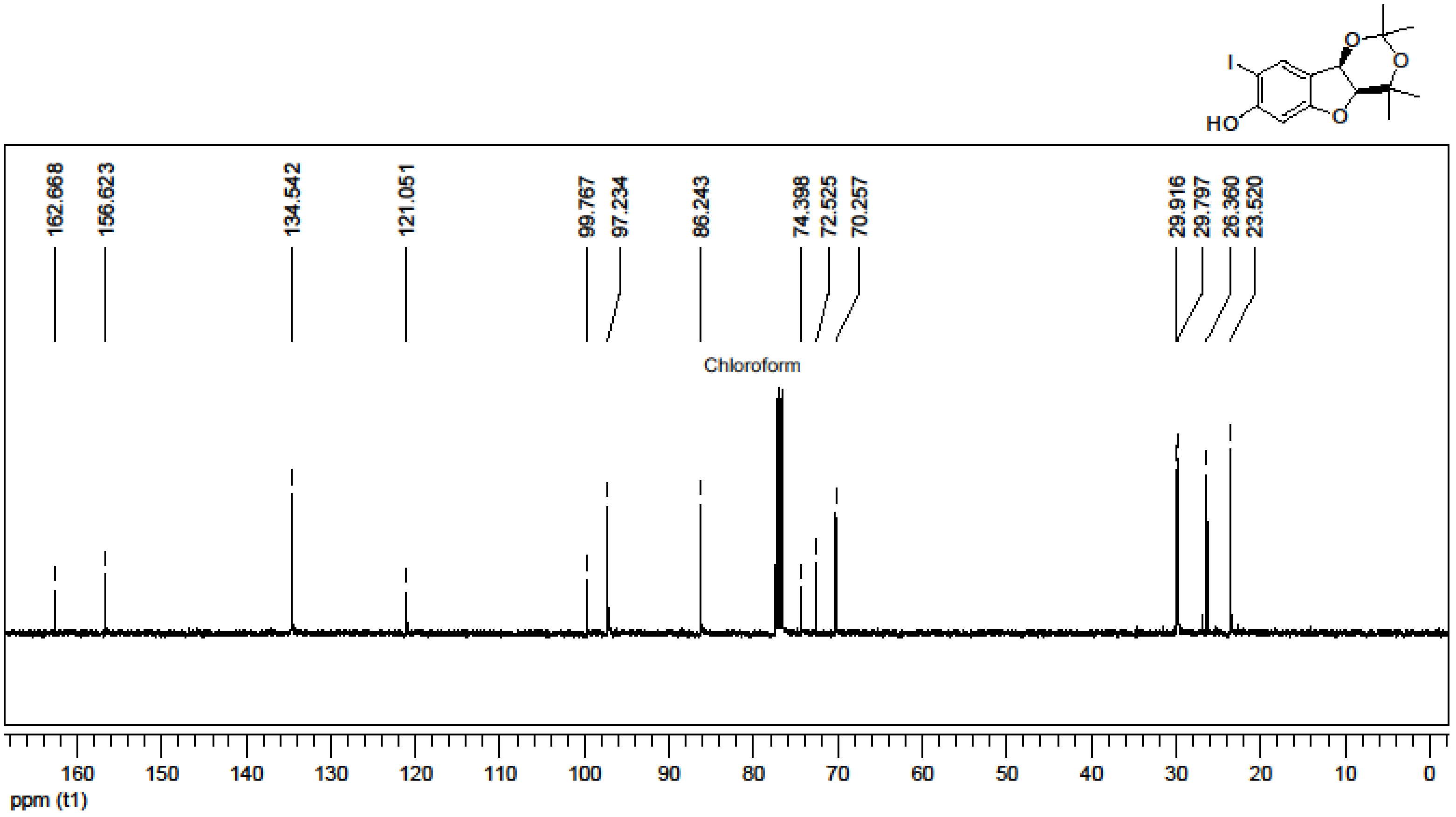
I
HO
O
O
O
162.668
156.623
134.542
121.051
99.767
97.234
86.243
74.398
72.525
70.257
29.916
29.797
26.360
23.520
Chloroform
160
150
140
130
120
110
100
90
80
70
60
50
40
30
20
10
0
ppm (t1)

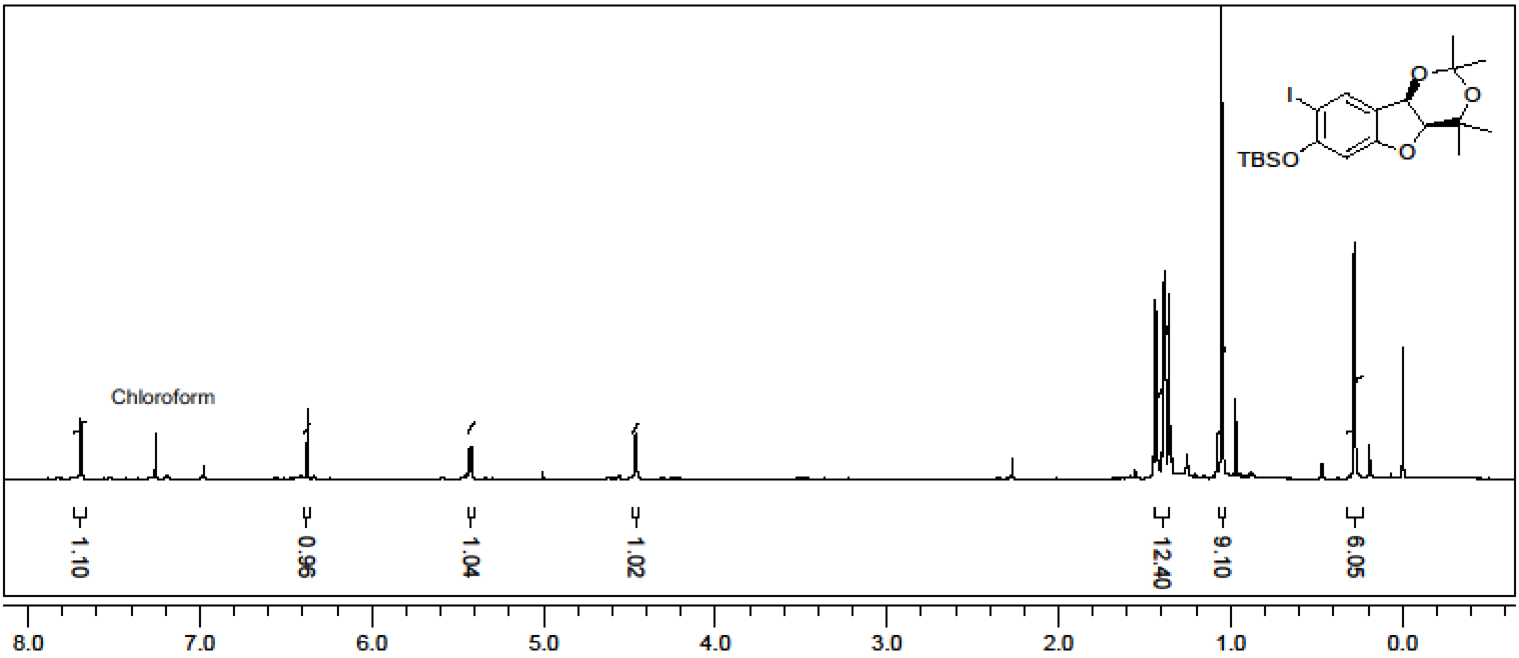
Chloroform
1.10
0.96
1.04
1.02
12.40
9.10
6.05
8.0
7.0
6.0
5.0
4.0
3.0
2.0
1.0
0.0
I
TBSO
O

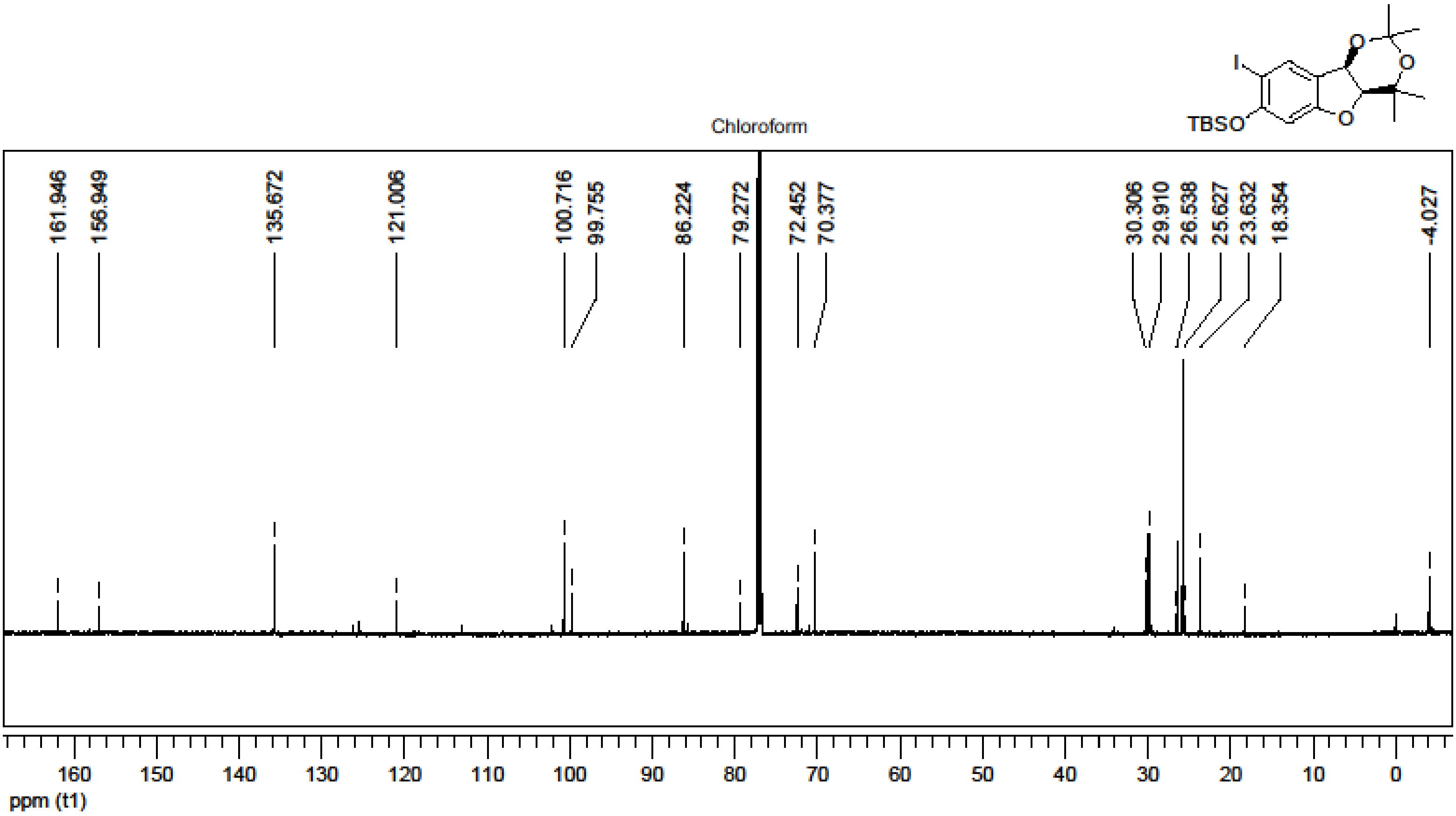
Chloroform
161.946
156.949
135.672
121.006
100.716
99.755
86.224
79.272
72.452
70.377
30.306
29.910
26.538
25.627
23.632
18.354
-4.027
160
150
140
130
120
110
100
90
80
70
60
50
40
30
20
10
0
ppm (t1)
I
TBSO
O

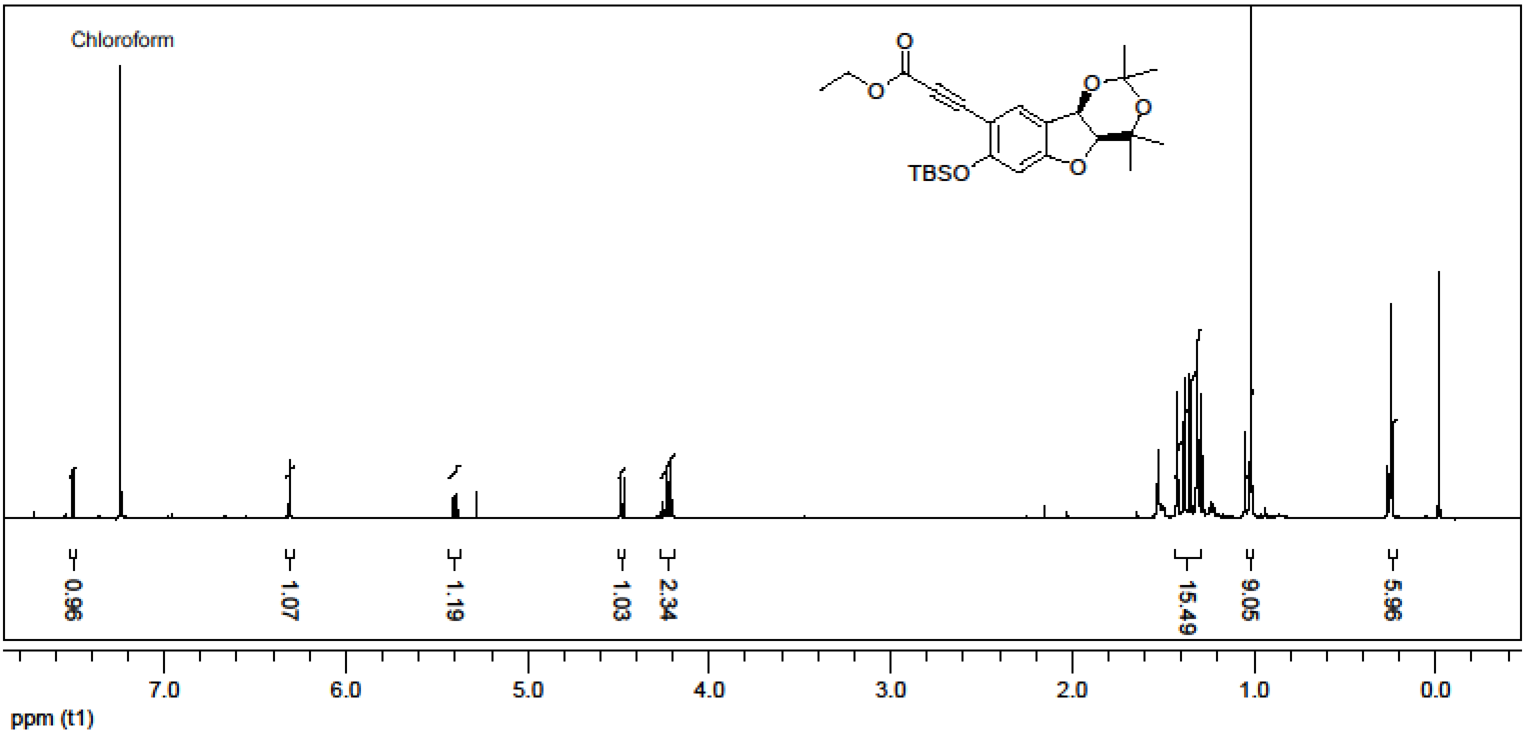
Chloroform
TBSO
0.96
1.07
1.19
1.03
2.34
15.49
9.05
5.96
7.0
6.0
5.0
4.0
3.0
2.0
1.0
0.0
ppm (t1)

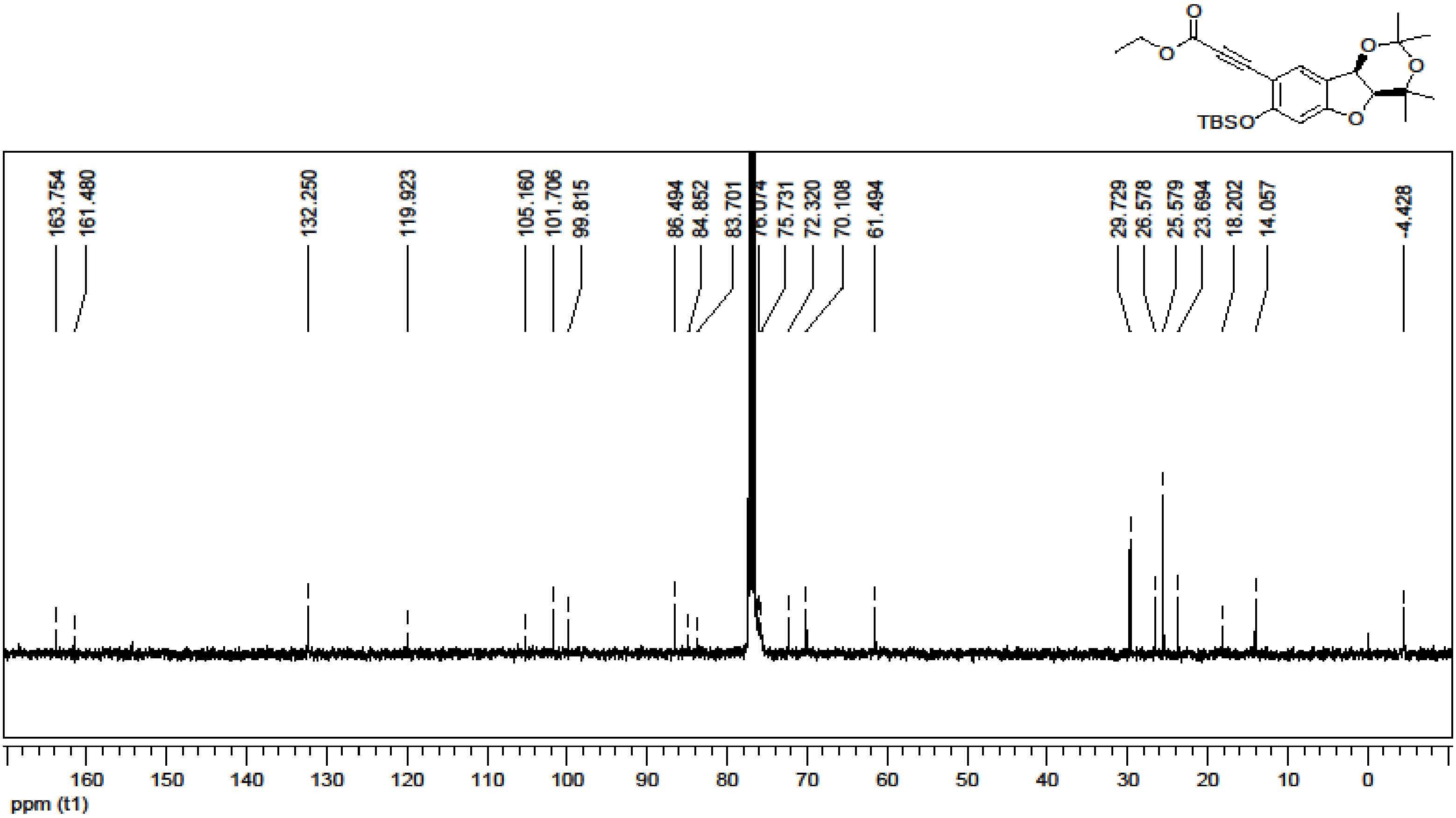
TBSO
163.754
161.480
132.250
119.923
105.160
101.706
99.815
86.494
84.852
83.701
76.074
75.731
72.320
70.108
61.494
29.729
26.578
25.579
23.694
18.202
14.057
-4.428
160
150
140
130
120
110
100
90
80
70
60
50
40
30
20
10
0
ppm (t1)

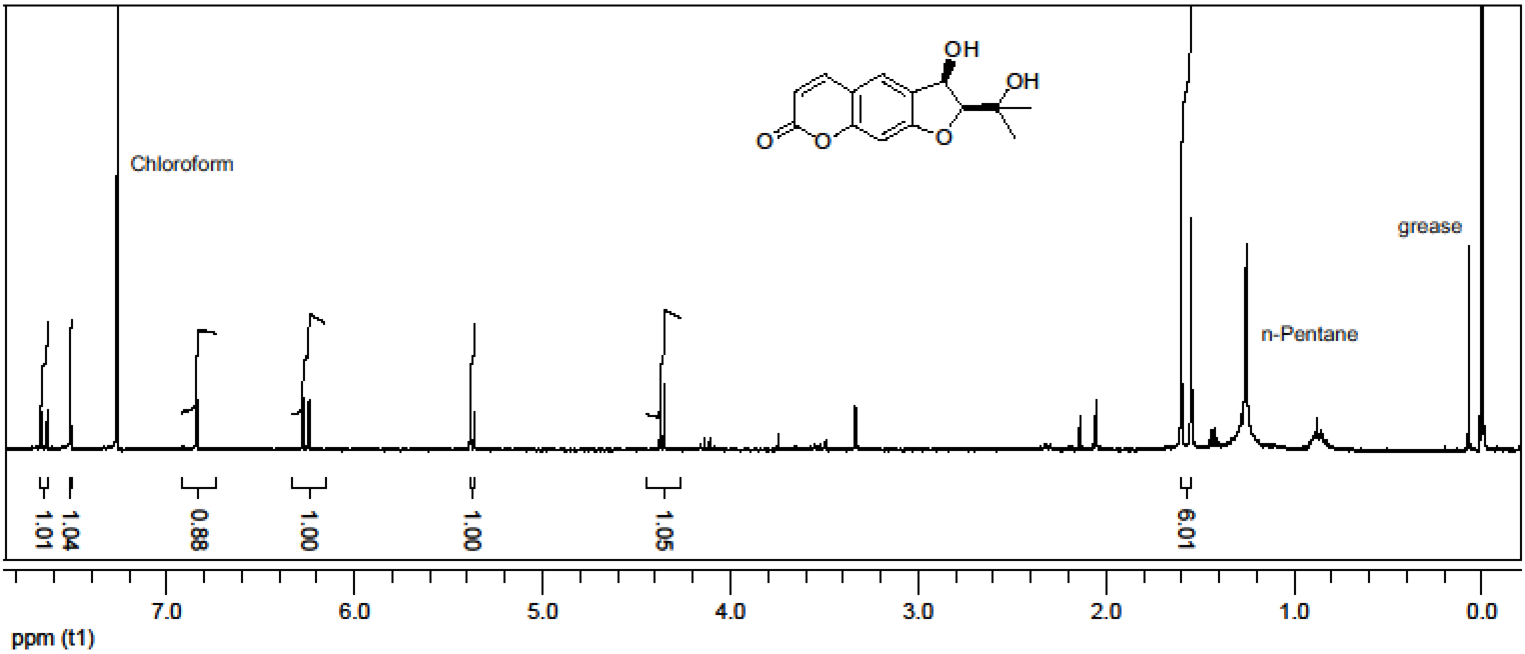
OH
OH
O
O
O
Chloroform
grease
n-Pentane
1.01
1.04
0.88
1.00
1.00
1.05
6.01
7.0
6.0
5.0
4.0
3.0
2.0
1.0
0.0
ppm (t1)

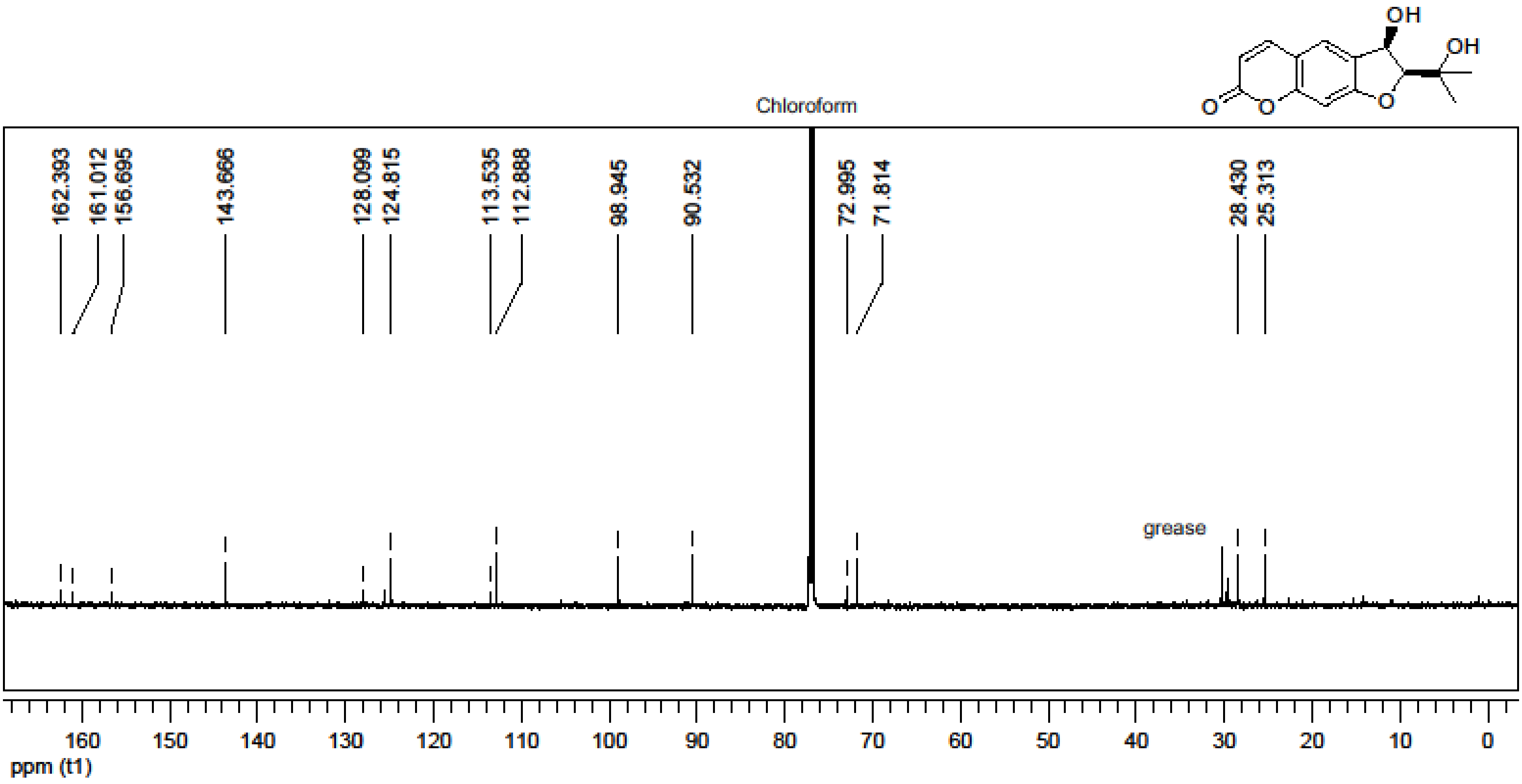
OH
OH
O
O
O
Chloroform
162.393
161.012
156.695
143.666
128.099
124.815
113.535
112.888
98.945
90.532
72.995
71.814
28.430
25.313
grease
160
150
140
130
120
110
100
90
80
70
60
50
40
30
20
10
0
ppm (t1)

Data file: D:\GONZO\JF\TB444RAD.D
Sample Info: Laufmittel: n-Heptan/EtOH 9:1;
Die Probe ist in DCM/LM gelöst.

Säule: DAICELAD.M
Säuleninfo: (250x4,6)mm
Operator: Analytik Labor AKEN

Injektion Time: 15:14:13
Injektion Date: 06.01.2012

Instrument Conditions:	At Start	At Stop
Temperature in°C:	30.0°C	30.0°C
Pressure in bar:	30.1	31.4
Flow in ml/min:	1.00	1.00

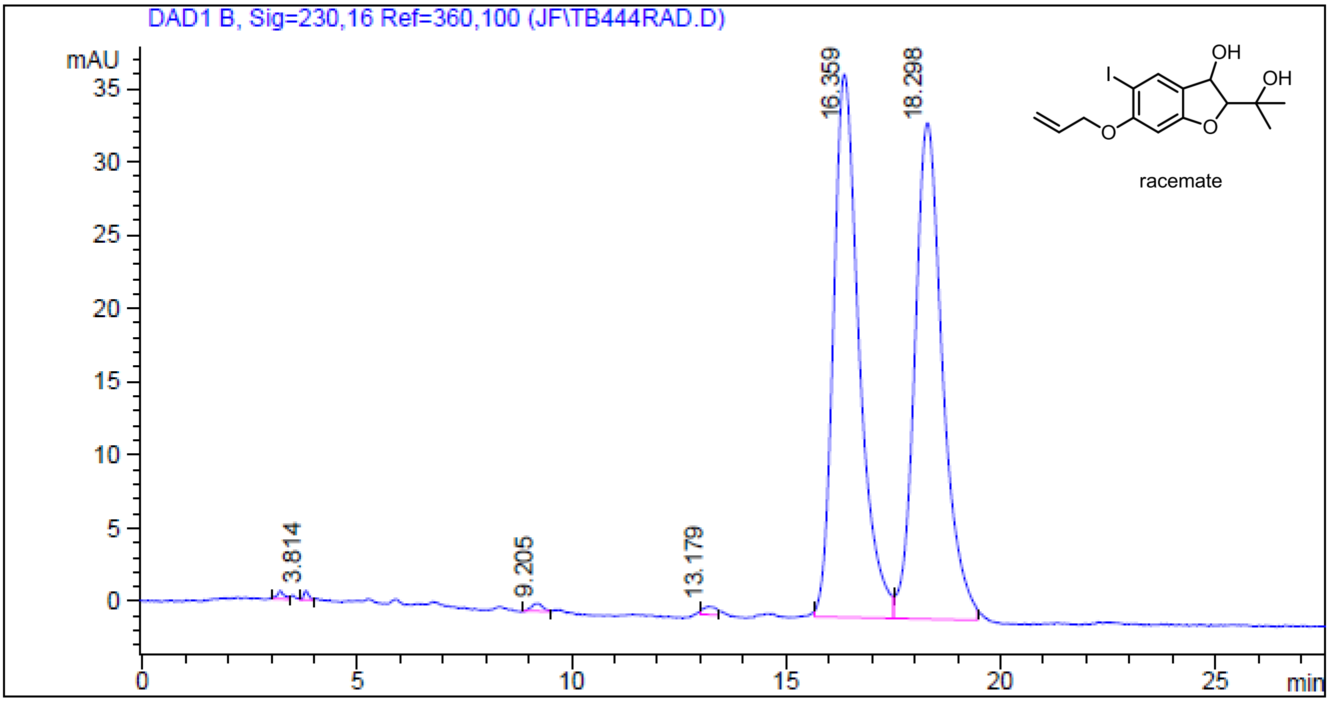

#	Ret. Time (min)	Width	Height (mAU)	Area (mAU*s)	Area %
1	3.21	0.16	0.56	6.61	0.21
2	3.81	0.12	0.62	4.96	0.16
3	9.20	0.24	0.53	9.45	0.30
4	13.18	0.26	0.56	11.60	0.37
5	16.36	0.62	37.08	1548.53	49.74
6	18.30	0.75	33.87	1532.40	49.22
Total				3113.55	100.00

Data file: D:\GONZO\JF\138K2AD.D
Sample Info: Laufmittel: n-Heptan/EtOH 9:1;
Die Probe ist in DCM/LM gelöst

Säule: DAICELAD.M
Säuleninfo: (250x4,6)mm
Operator: Analytik Labor AKEN

Injektion Time: 11:12:10
Injektion Date: 18.01.2012

Instrument Conditions:	At Start	At Stop
Temperature in°C:	30.0°C	30.0°C
Pressure in bar:	23.3	24.2
Flow in ml/min:	1.00	1.00

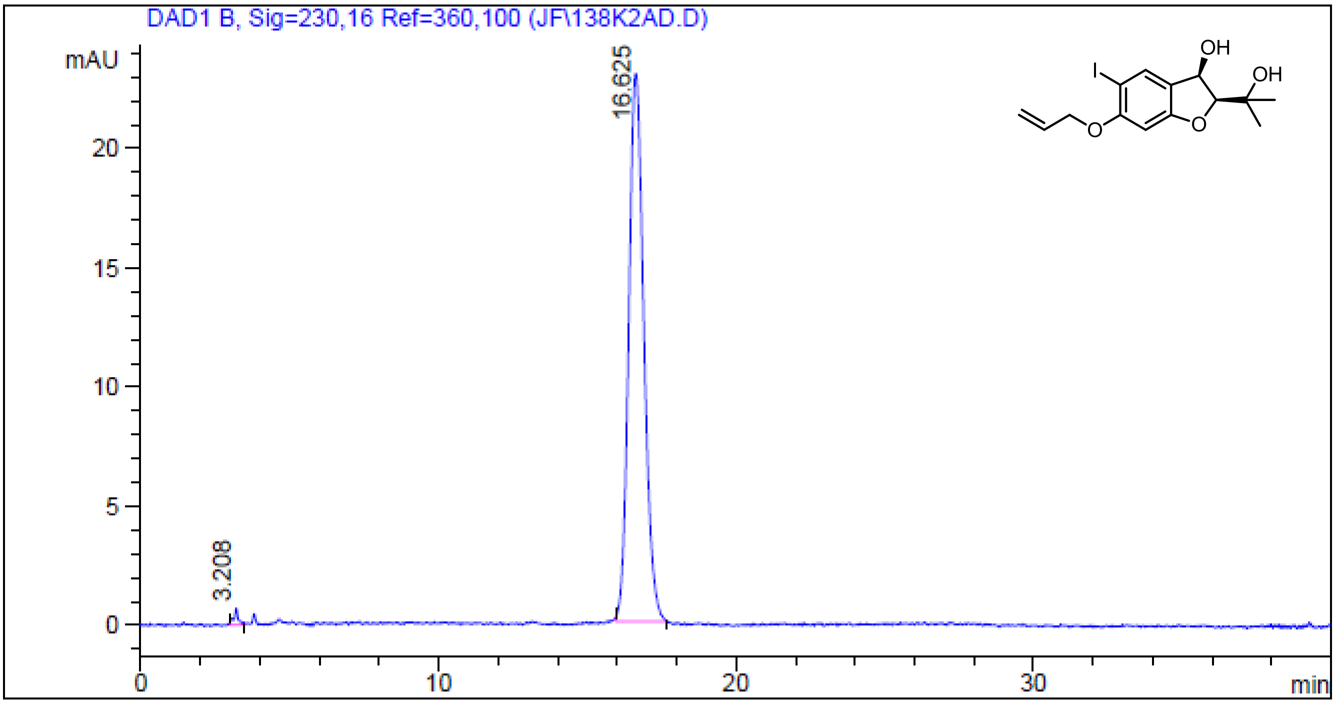

#	Ret. Time (min)	Width	Height (mAU)	Area (mAU*s)	Area %
1	3.21	0.14	0.70	7.31	0.89
2	16.63	0.54	22.99	812.30	99.11
Total				819.61	100.00
